# Genetic and phenotypic variation along an ecological gradient in lake trout *Salvelinus namaycush*

**DOI:** 10.1186/s12862-016-0788-8

**Published:** 2016-10-19

**Authors:** Shauna M. Baillie, Andrew M. Muir, Michael J. Hansen, Charles C. Krueger, Paul Bentzen

**Affiliations:** 1 Marine Gene Probe Lab, Department of Biology, Dalhousie University, 1355 Oxford Street, PO Box 15000, Halifax, NS B3H 4R2 Canada; 2 Great Lakes Fishery Commission, 2100 Commonwealth Boulevard, Ann Arbor, MI 48105 USA; 3 U.S. Geological Survey, Great Lakes Science Center, Hammond Bay Biological Station, 11188 Ray Road, Millersburg, MI 49759 USA; 4 Department of Fisheries and Wildlife, Center for Systems Integration and Sustainability, Michigan State University, East Lansing, MI 48824-1222 USA

**Keywords:** Phenotype, Multiple coinertia analysis, Geometric morphometrics, Microsatellite DNA, Lake Superior, Intraspecific variation, Adaptation

## Abstract

**Background:**

Adaptive radiation involving a colonizing phenotype that rapidly evolves into at least one other ecological variant, or ecotype, has been observed in a variety of freshwater fishes in post-glacial environments. However, few studies consider how phenotypic traits vary with regard to neutral genetic partitioning along ecological gradients. Here, we present the first detailed investigation of lake trout *Salvelinus namaycush* that considers variation as a cline rather than discriminatory among ecotypes. Genetic and phenotypic traits organized along common ecological gradients of water depth and geographic distance provide important insights into diversification processes in a lake with high levels of human disturbance from over-fishing.

**Results:**

Four putative lake trout ecotypes could not be distinguished using population genetic methods, despite morphological differences. Neutral genetic partitioning in lake trout was stronger along a gradient of water depth, than by locality or ecotype. Contemporary genetic migration patterns were consistent with isolation-by-depth. Historical gene flow patterns indicated colonization from shallow to deep water. Comparison of phenotypic (*Pst*) and neutral genetic variation (*Fst*) revealed that morphological traits related to swimming performance (e.g., buoyancy, pelvic fin length) departed more strongly from neutral expectations along a depth gradient than craniofacial feeding traits. Elevated phenotypic variance with increasing water depth in pelvic fin length indicated possible ongoing character release and diversification. Finally, differences in early growth rate and asymptotic fish length across depth strata may be associated with limiting factors attributable to cold deep-water environments.

**Conclusion:**

We provide evidence of reductions in gene flow and divergent natural selection associated with water depth in Lake Superior. Such information is relevant for documenting intraspecific biodiversity in the largest freshwater lake in the world for a species that recently lost considerable genetic diversity and is now in recovery. Unknown is whether observed patterns are a result of an early stage of incipient speciation, gene flow-selection equilibrium, or reverse speciation causing formerly divergent ecotypes to collapse into a single gene pool.

**Electronic supplementary material:**

The online version of this article (doi:10.1186/s12862-016-0788-8) contains supplementary material, which is available to authorized users.

## Background

Sympatric ecological speciation is a process by which a segment of a population develops some level of reproductive incompatibility with other population members in the absence of geographic barriers and complete cessation of gene flow [1]. Today, advances in genomics [2] and epigenetics [3, 4] have enabled testing of predictions generated by novel hypotheses on mechanisms underlying this evolutionary process. Sympatric ecological speciation has been summarized into four main steps from an evolutionary genomics perspective: first, an initially panmictic population experiences a new environment; second, disruptive natural selection causes divergence of functional phenotypic traits and adaptive loci while gene flow at neutral loci continues; third, gene flow at neutral loci becomes partially restricted; and fourth, complete reproductive isolation and speciation [1]. The first stage involves character release where individuals of a species are exposed to new ecological opportunities [5]. This character release stage typically shows high levels of within-group phenotypic variance where phenotypic responses to the environment may be mainly epigenetic (i.e., due to gene expression). The second stage describes how ecological opportunity can give rise to resource polymorphism where functional traits may become genetically accommodated, or hard-wired in the genome [6–8]. In the third stage, given a degree of environmental stability, ecologically and phenotypically divergent intraspecific forms, or ecotypes, with partially restricted gene flow may arise. In the fourth and final stage, reproductive isolation evolves among morphs leading to speciation. At each stage, a collapse into panmixis can occur when divergent selection is relaxed [5]. This relaxation can be brought about by environmental change in which a heterogeneous environment (e.g., Enos Lake sticklebacks [9]) or diverse food sources are homogenized, as Darwin’s finches experienced after seed type homogenization [10]. Alternatively, changes in species abundance resulting from local extirpations or release from predation pressure or both (e.g., Lake Huron coregonids *Coregonus* spp. [11] or shifts in community structure (e.g., Scandinavian coregonids [12]) could also lead to relaxed divergent selection. Additionally, hybridization of weakly diverged conspecifics can result in high within-group phenotypic variability [5]. Therefore, although the stage of speciation may be assessed, the direction of evolutionary change cannot be discerned unless time-series data are available to assess directionality of selection and divergence.

Understanding whether gene flow is partially restricted along an ecological gradient can provide evidence of the prime drivers of selective divergence and maintenance of ecotypes [13–16]. Thus, studies of niche divergence along environmental gradients can be important to species conservation and understanding of the interaction between human-mediated disturbance and evolution of wild populations. In most aquatic environments, water depth is an especially important gradient that correlates with many environmental variables (e.g., hydrostatic pressure, light intensity, light quality, temperature, pH, or oxygen concentration). Phenotypic and reproductive divergence along water depth gradients has been described in Lake Victoria cichlids [17, 18] and coregonids in Europe and North America [19–22]. Thus, studies that compare neutral genetic and phenotypic divergence, *Pst* (term coined by Leinonen et al. [23]), can help ascertain the relative influence of selection on genes and random genetic drift on population differentiation [23]. *Pst* and neutral genetic divergence (as measured using *Fst*) can be compared among and within groups to assess the stage of ecological speciation. Theoretically, if *Pst* is greater than *Fst*, then the phenotypic trait in question is interpreted to exceed levels of divergence based on neutral expectations, and is therefore under selection [23].

Here, we focus on neutral genetic and phenotypic variation in lake trout *Salvelinus namaycush*, a species believed to have developed partially reproductively isolated ecotypes in sympatry (e.g., [24]). Differentiation among lake trout ecotypes has been associated primarily with differences in traits related to trophic resource partitioning (e.g., cranioskeletal features) and locomotion (e.g., fin length) [25–30]. In Lake Superior, four ecotypes have been described: lean, humper, siscowet, and redfin [25]. The lean ecotype is considered the ancestral form, in part because it is the most widely distributed across North America [31]. Relative to other ecotypes, lean lake trout typically use relatively shallow water (<80 m depth) and have adaptations for sustained swimming similar to that required in fluvial environments. Siscowet are the most abundant ecotype [32, 33], typically occupy deep water (>50 m), and have much higher fat content than leans. Historically, lean and siscowet would have fed differentially on a suite of cisco species (*Coregonus* spp.; [32]). Since the introduction of non-native forage fishes and shifts in the native forage base in Lake Superior, adult lean and siscowet lake trout now prey on similar shallow water diet items, but in differing proportions [26, 32, 33]. Humper lake trout live on isolated offshore reefs (or “humps”) or on steep sloping banks surrounded by deep water [34], and are intermediate in fat content between lean and siscowet lake trout [35]. The diet of humpers has not been described in detail; however, a humper-like ecotype from Lake Mistassini, Quebec had a diet rich in pelagic opossum shrimp *Mysis relicta* [28], which are abundant in Lake Superior. The redfin only recently has been described as a distinct ecotype in Lake Superior and is the largest bodied of the ecotypes [25]. Little is known about the diet of redfins, but they occupy deep water (>80 m) and likely have diets similar to that of siscowet.

Our main objectives were two-fold: 1) determine if contemporary lake trout genetic, morphological, and life history trait variation are divergent along a water depth gradient; and 2) determine the stage of ecological divergence (e.g., panmixis, restricted gene flow) at which lake trout exist today in Lake Superior. To accomplish this, we directly compared genotype to phenotype, assessed gene flow, and quantified divergence of phenotypic traits of lake trout among water depth strata. Historically, water depth was considered a primary axis for lake trout ecotype divergence (e.g., [36]). This hypothesis was supported by breeding experiments on physiological traits related to maintaining their position in the water column and capability to move among depths [35, 37–39], and associations of ecotypes with depth of capture [40]. Due to the recently documented overlap in morphology [25] and genetic diversity [41] among putative ecotypes, lake trout may have collapsed into a single gene pool and ecological axes of divergence for lake trout may have diminished. If phenotypic variation is divergent along an axis in which gene flow is partially restricted, then we confirm the persistence of ecological opportunities that can maintain or promote divergence. Such information will be relevant for documenting intraspecific biodiversity [42] in the largest freshwater lake in the world, and for the re-establishment in other Laurentian Great Lakes of a native fish that has lost considerable genetic diversity due to human disturbance [36, 43, 44].

## Methods

### Sampling

**Fig. 1:**
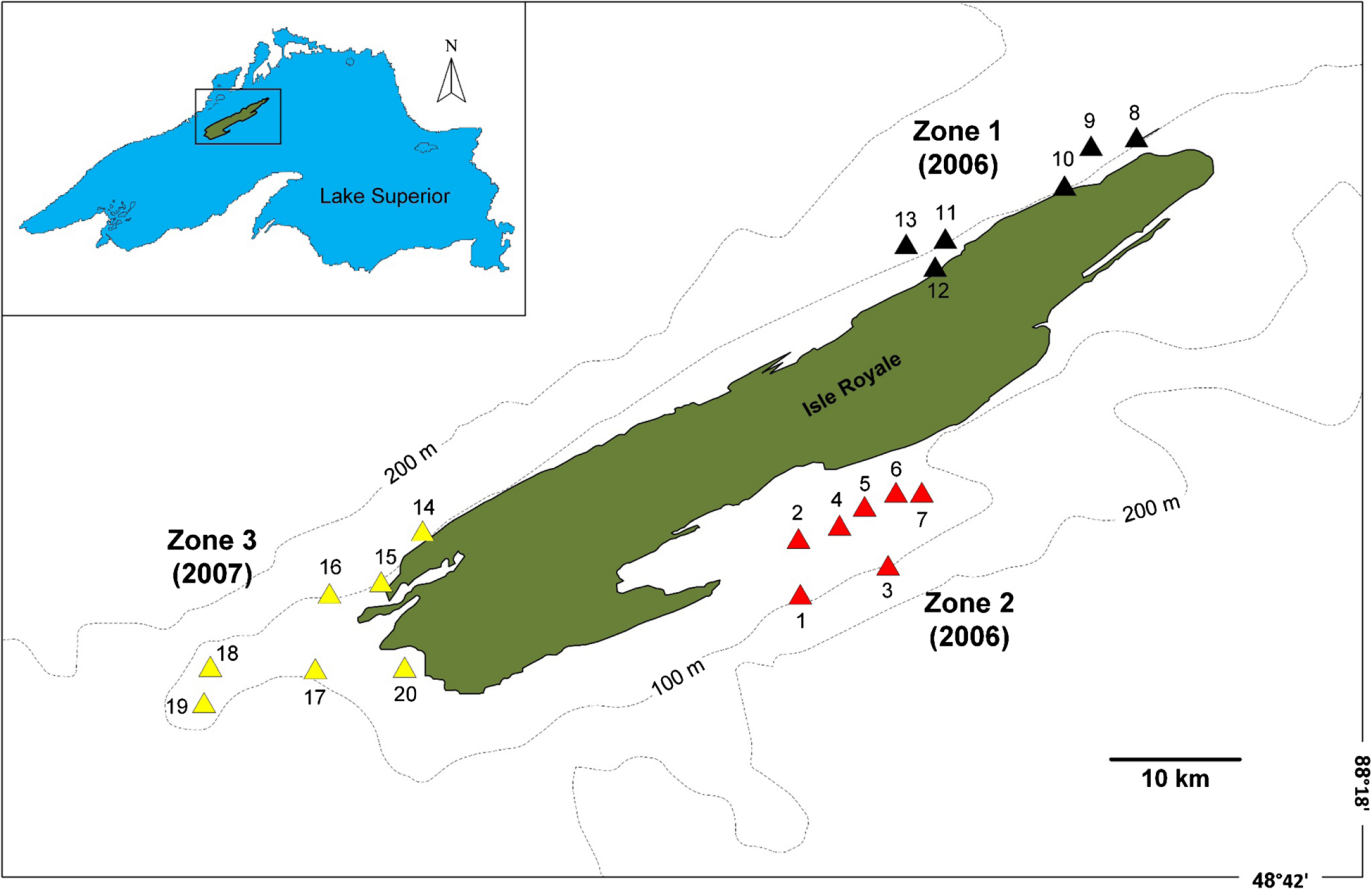
Map of study area. Lake trout sampling sites (*solid triangles*) at three geographic zones around Isle Royale, Lake Superior. Sampling year at each zone is indicated. This figure has been modified with permission from Muir et al. [25]

Lake trout were sampled at Isle Royale, Lake Superior (N 48 °00; W 88 °50) during August 2006 and 2007 (Fig. 1) by Muir et al. [25]. Gillnet sampling was conducted at 20 sites across three geographic zones around Isle Royale: Zone 1, northwest tip of the island; Zone 2, central part of east side of the island; Zone 3, southern tip of the island. Gillnet sets (one set per site, each deployed on bottom for 24 h) were distributed equally across three depth strata: shallow (<50 m), intermediate (50–100 m), and deep (>100 m) [25]. Siscowet were the most abundant ecotype sampled and identified by Muir et al. [25] (53.9 %, *N* = 319/592), then lean (23.9 %, *N* = 142/592), humper (12.1 %, *N* = 72/592), and redfin (10.1 %, *N* = 60/592). Considering the relative abundance of lake trout within each water depth stratum, leans were the most abundant ecotype in shallow water (60.3 %, *N* = 76/126), and only comprised 12.4 % (*N* = 39/315) and 17.9 % (*N* = 27/152) of lake trout caught in intermediate and deep water, respectively. Siscowet dominated intermediate (58.4 %, *N* = 184/315) and deep water depths (61.8 %, *N* = 94/152), with 32.5 % (*N* = 41/126) in <50 m shallow water. Humper and redfin, caught mainly in >80 m of water, were less abundant than lean and siscowet within each depth strata (ranging from 3.2 to 16.4 % for humpers and 3.9–15.6 % for redfins). All four ecotypes were present in similar proportions in all three geographic zones: siscowet (range = 51–56 %), leans (range = 21–29 %), humpers (range = 10–14 %), and redfins (range = 4–14 %).

### Morphological measurements

**Fig. 2:**
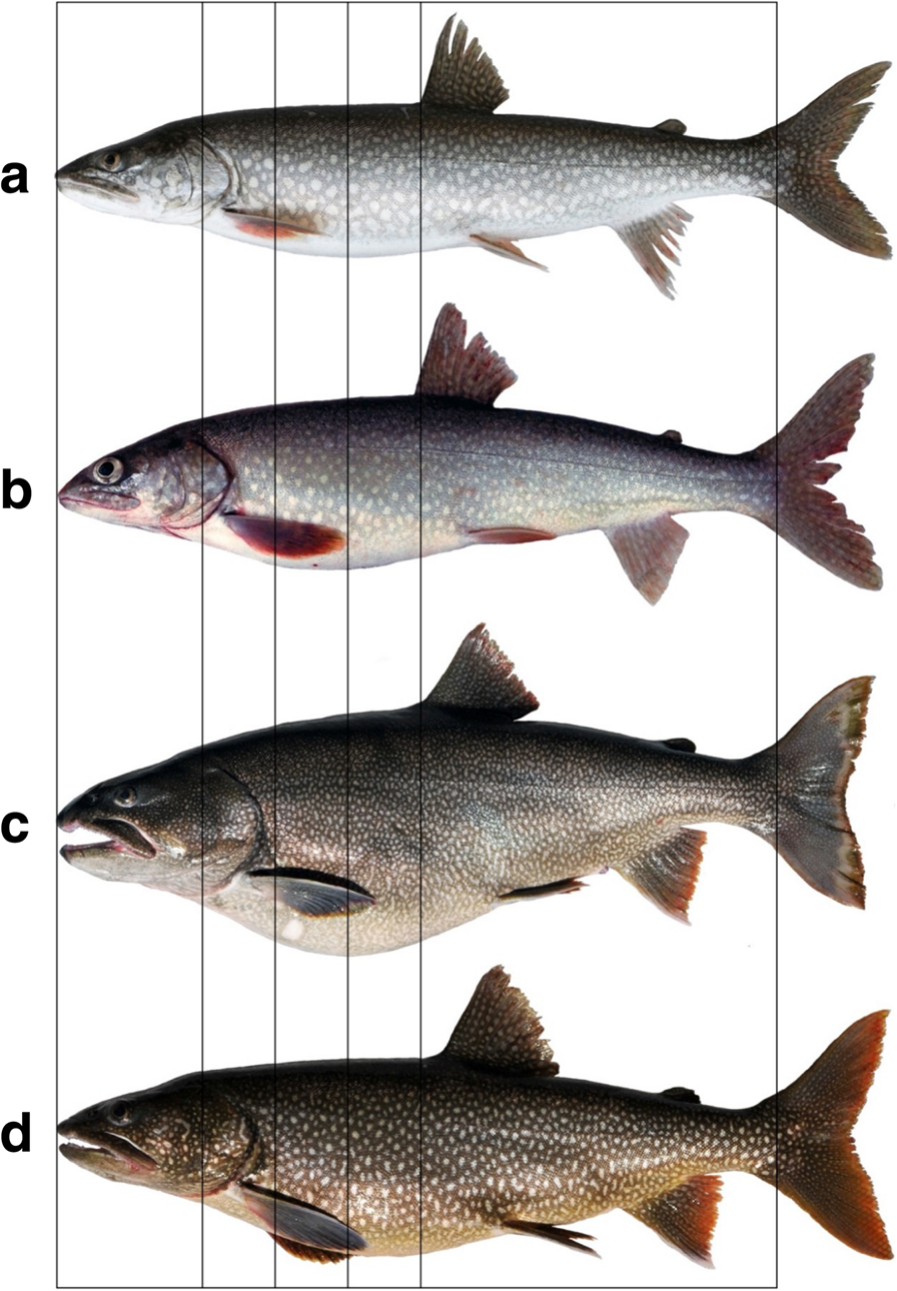
Illustrations of four lake trout ecomorphs from Isle Royale, Lake Superior. Photographs of lean (**a**), humper (**b**), siscowet (**c**) and redfin (**d**) are shown. The *vertical lines* provide a sense for relative sizes and positions of key anatomical features, such as head size and fin insertion and lengths among the ecomorphs. Note that the four fish have been size-scaled to the same focal length

To identify four lake trout ecotypes, variation in head and body shape was quantified by Muir et al. [25] using geometric morphometric methods [27, 45, 46] on whole body digital images, as implemented in the Thin Plate Spline suite (State University of New York at Stony Brook; http://life.bio.sunysb.edu/morph): lean, humper, siscowet, and redfin (Fig. 2). Eight landmarks (homologous points) and 20 semi-landmarks (used to compare homologous curves) defined head shape, and 4 semi- and 16 homologous landmarks defined body shape for 593 adult fish (i.e., >430 mm, see [27]; Additional file 1). Landmark data were used to obtain size-adjusted partial warp scores for each fish for head and body shape in two separate analyses [25]. Principal components analysis (PCA) was used to reduce dimensionality of warp scores to four principal components (*PC*s) for head and body data sets [25]. Subsequently, *PC*s were used by Muir et al. [25] to identify morphological groups using a Bayesian clustering package implemented in R (MCLUST; [47]). The first four *PC*s in an ordination of body shape accounted for 66 % of the variation and the first four *PC*s in an ordination of head shape accounted for 72 % of the variation [25]. *P*Cs beyond four only increased the amount of explained variation by less than 5 % each and did not add any additional discriminatory power to the MCLUST models, therefore, on the basis of parsimony were omitted from the analysis. Morphological groups were then identified by a combination of statistical and visual methods to achieve a consensus identification for each individual [25].

Additionally, linear measurements for eight putatively functional traits linked to feeding and locomotion measured by Muir et al. [25] were used in this study to examine signatures of phenotypic selection across habitats: caudal peduncle depth (CPD), caudal peduncle length (CPL), head length (HLL), maxilla length (MXL), orbital length (OOL), pectoral fin length (PCL), pelvic fin length (PVL), and pre-orbital length (POL) (See Additional file 1). Buoyancy was measured as an adaptive trait, because it is positively correlated with body lipid content and capture depth [27]. As previously described in Muir et al. [25], percent buoyancy was calculated as the difference of the weight-of-fish-measured-in-air minus the weight-of-fish-measured-in-water, divided by weight-of-fish-measured-in-air times 100 [25]. Linear and buoyancy measurements for each fish were log_10_ transformed and then regressed against standard length (SL). The studentized residuals were used as size-adjusted variables in subsequent analyses.

### Life history trait estimation

Five life history traits were calculated in a concurrent study by Hansen et al. [48] on the same lake trout samples represented in this study. Annuli from transverse thin sections of dried sagittal otoliths were counted to estimate the age for each specimen. Nonlinear mixed-effects models were used to estimate growth parameters for individual fish, based on biological intercept back-calculations of growth histories of individual fish [49]: age-at-time-zero (*t*_0_ = years; incubation time of embryos from fertilization to hatching); length-at-time-zero (*L*_0_ = mm; length at emergence from the egg); instantaneous growth rate (*K* = 1/year) at which fish length approaches the theoretical maximum length (*L*_max_), herein referred to as asymptotic length; early annual growth rate (*ω* = *L*_max_ × *K* = mm/year; [50]). The lake trout specific biological intercept was based on equations describing relationships between length, age in days, and sagittal otolith width of age-0 estimated by Bronte et al. [51] where age-0 lake trout sagittal otolith radius = 0.137 mm and length = 21.7 mm [52]. Detailed methods for these life history analyses are given by Hansen et al. [48].

### DNA extraction and microsatellite amplification

Total genomic DNA was extracted using a glass-milk binding protocol [53]. Eighteen microsatellite loci were genotyped using a variety of previously published primer combinations (see Additional file 2). Polymerase chain reactions (PCR) were conducted using 0.5 μL of 10x Thermopol reaction buffer, 200 μmol/L of dNTPs, 2 pmol of the forward and reverse primer, 0.2 units of *Taq* DNA polymerase, and 1 μL of DNA in 5 μL volumes. PCR conditions consisted of 95 °C for 5 min, 25–30 cycles of 94 °C for 30 s, locus specific annealing temperature (50–62 °C) for 30 s, 72 °C for 1 min, and 72 °C for 3 min. Amplicons were visualized on LI-COR Biosciences (Lincoln, NE, USA) DNA Analyzers with 6 % denaturing polyacrylamide gels. Scoring was performed by hand using a standard ladder (100–350 bp) and scores verified using two positive controls on every gel.

### Statistical analyses

#### Sources of genetic structure

Three potential sources of group structure were recorded for each fish: morphology (ecotype), geographic location around Isle Royale (zone), and water depth stratum. Total genetic variation was partitioned with a hierarchical analysis of molecular variance (AMOVA) based on 10,000 permutations in ARLEQUIN. Several AMOVAs were performed to determine the variable that most strongly explained genetic organization among groups using the *Fct* statistic for main effects of ecotype, zone, and water depth stratum, as well as all two-way combinations of those variables.

#### Genetic diversity

MICROCHECKER 2.2.3 [54] assessed scoring errors and the presence of null alleles. CREATE 1.37 [55] converted file formats from our original raw data file. Hardy Weinberg Equilibrium (HW) and linkage disequilibrium were tested using ARLEQUIN 3.5 [56]. All multi-test adjustments [e.g., HW, linkage disequilibrium) were based on a sequential goodness of fit metatest using the program R [57] package ‘SGoF’ 3.8 [58], except where stated otherwise. FSTAT 2.9.3.3 [59] and ARLEQUIN measured the number of alleles (*A*), allelic richness (*Ar*), the number of private alleles, and observed and expected heterozygosity (*Ho* and *He*_,_ respectively). Private allelic richness (*PAr*), the number of private alleles standardized by sample size for each group, was calculated in HP-RARE [60], inbreeding coefficients, *Fis* [61], were calculated using FSTAT, and significance of *Fis* departure from HW was tested using 10,000 permutations at α = 0.05.

#### Genetic structure within and among groups

Principal coordinates analysis (PCOA) in GENALEX 6.5 [62] was used to visualize genotypic distribution of individual fish in multivariate space. Several Bayesian clustering analyses with the admixture model with *a priori* assumptions based on ecotype, zone, and water depth stratum in STRUCTURE 2.3.4 [63–65] determined the number of genetic groups, *k*. For each analysis, 10 independent runs were conducted for each value of *k* from *k* = 1 to *k* = 10 with burn-in length of 2.5 × 10^5^, followed by 1.0 × 10^6^ randomization step. Both the Evanno et al. [66] and Pritchard et al. [63] methods, as well as an estimation of when posterior probabilities for *k* plotted against *k* reached a plateau [63], estimated the most probable *k* from the STRUCTURE results. Delta *k* (∆*k*) and the estimated natural log probability of *k* (ln *P*(*k*)) [66] were generated in STRUCTURE HARVESTER Web 0.6.92 [67]. As an indicator of differentiation among groups, 10,000 permutations in ARLEQUIN compared pairwise *Fst* [68]. ‘DEMEtics’ 0.8–2 [69] package was implemented in R to obtain Jost *D* [70] and its significance values (*P*) [71] using 10,000 bootstrap re-samplings. The Bonferroni correction for Jost *D* was performed using ‘DEMEtics’ [69]. Mantel tests assessed correlations of genotype with water depth strata and geographic distance, as implemented in GENALEX with 9999 permutations at an alpha level of *P* <0.05 for significance tests. Note that the original study design involved fish sampled at three water depth strata (<50 m, 50–100 m, and >100 m). More than three points (water depth strata) are required to perform a Mantel test in GENALEX. Thus, for this analysis only, the dataset was further subdivided into six water depths (40, 50, 80, 90, 100, and >120 m) based on maximum capture depth of net sets of individual fish. These depth categories while uneven (lacking 60 and 70 m, reflect the distribution of net depths in the data set. To test for isolation-by-distance along a geographic distance axis, a second Mantel test was performed on genotype against geographic distance among the 20 sampling sites.

Historical gene flow among the three original water depth strata (<50 m, 50–100 m, >100 m) was estimated using the maximum-likelihood approach in MIGRATE 3.03 [72, 73]. MIGRATE analyses were performed assuming an infinite allele model (IAM) and the parameters that follow: 10 short chains with a sampling increment of 100 where 500 genealogies are sampled; three long chains with a sampling increment of 1000 where 5000 genealogies are sampled. We discarded 10,000 genealogies at the beginning of each chain as burn-in, and averaged maximum-likelihood estimates over five independent runs.

#### Phenotypic trait variation among groups

To assess clinal relationships in morphological and life history traits, data was plotted graphically with their standard deviations. One-way ANOVA was performed for each trait by the appropriate independent grouping variable, e.g., water depth, and pairwise post hoc Fisher’s least significant difference (LSD) tests were conducted using SPSS 20.0 (IBM Corp. Released 2011. IBM SPSS Statistics for Windows, Version 20.0. Armonk, NY: IBM Corp.).

#### Genetic associations with phenotypic trait variation

Phenotypic variance (*Pst*) estimates on morphological and life history traits among the three original water depth strata were compared to *Fst* [74] estimates. *Pst* is a distance matrix analogue to *Fst* [23]. Phenotype-environment associations are assumed to evolve under divergent natural selection, while DNA sequences such as microsatellite loci evolve neutrally [23, 75], have an additive genetic basis (where genes contribute a ‘fixed’ phenotypic value), and presumably are under stabilizing selection [22]. On the other hand, divergent or directional selection of a phenotypic trait is implied when *Pst* is greater or less than *Fst*.

Among- (*Pst*) and within-group (*r*ii) phenotypic variance was estimated for morphological traits by calculating genetic relationship matrices (*R-*matrix estimates) in program RMET 5.0 [76–78]. Phenotypic distances were adjusted for sample size in RMET, and we used a heritability score of 1.0 as recommended by Leinonen et al. [23]. The program output contains an *R*-matrix (a variance-covariance matrix), a *D* ^*2*^-matrix (producing *D* ^*2*^ estimates based on a Mahalanobis distance), and a global *Pst*-value (global *Fst*-value analogue and measure of overall variance across all groups). For pairwise comparisons of phenotypic to genetic distances among different water depth strata, we used the *D* ^2^ estimates (and their standard error estimates as calculated in RMET) because no heritability estimation was required. *Fst* for microsatellite markers was calculated with bootstrapped 95 % confidence intervals using the ‘diveRsity’ [79] package in program R [57]. Subsequently, and for direct comparison with *Pst*-values, the upper and lower confidence limits around *Fst* were converted to standard error (*SE*) using the following equation: *SE* = (upper limit – lower limit)/3.92. If within-group phenotypic traits showed greater variance at intermediate water depths, hybridization was considered as an explanation [80]. On the other hand, if variance increased with an increase in water depth, it was interpreted as divergent selection and character release in early stages of sympatric divergence [5].

## Results

### Sources of genetic structure

Among ecotype, zone, and depth stratum, only depth stratum explained (AMOVA *Fct* = 0.01) molecular variance among groups (Table 1). Ecotype, zone, or any combination of these variables with each other and with depth stratum did not reveal any significant genetic structuring. Overall, the highest levels of among group genetic variation was attributable to depth stratum.

**Table 1:** Analyses of molecular variance (AMOVA) for Isle Royale lake trout

Source of variation (standard AMOVA)	*Fct*	*d.f.*	Sum of squares	Variance components	Percentage of variation	*P*-value
Ecotype	0.002	3	9.33	0.006	0.20	0.480
Zone	−0.002	2	2.35	−0.005	−0.17	0.889
**Stratum**	**0.010**	**2**	**15.2**	**0.027**	**0.99**	<0.001*
Ecotype and zone	−0.002	11	23.8	−0.005	−0.18	0.862
Ecotype and stratum	0.004	10	29.7	0.013	0.49	0.368
Zone and stratum	0.007	8	26.2	0.020	0.71	0.207

Fish were grouped by ecotype (lean, humper, siscowet, and redfin), geographic location (Zone 1, 2, and 3), and water depth strata (<50 m, 50–100 m, and >100 m). AMOVAs were based on 18 polymorphic microsatellite loci and levels of significance were extracted after 10,000 permutations as implemented in ARLEQUIN. The strength of the source of genetic partitioning can be ranked from the highest (in bold) to lowest significant *Fct* value (genetic distance among groups), the measure of among group differentiation. Asterisks mark significant tests at alpha = 0.05

Because depth stratum was the strongest variable associated with group genetic structure according to AMOVA, subsequent genetic analyses were performed with fish samples grouped by water depth, and not by ecotype or zone, except where stated otherwise. Also, Zone 3 was excluded from analyses due to the interaction between zone and depth stratum, except where stated otherwise.

### Genetic diversity

Most loci (all individuals pooled) showed moderate levels of variation with the number of alleles ranging from three to 25 and averaging 11.5 (Additional file 3). Possible null alleles were detected in one, *Sfo*334, of 18 loci consistently across ecotypes and depth strata according to MICROCHECKER. After sequential goodness of fit correction [58], 20 pairs (13 %) of loci had significant linkage disequilibrium. For analyses dependent on assumptions of HW and informative loci (e.g., STRUCTURE, PCOA) the locus Sfo334 was excluded due to null alleles, and *SalD*39 and *OneU*9 were excluded due to low *He*. No *Fis* estimates deviated significantly from HW when the dataset was divided by water depth strata (Table 2), zone, or ecotype (Additional file 4). Similarly, *He* and *Ar* did not differ (*P* = 0.43) among depth strata. Lake trout within the intermediate depth stratum (50–100 m) had the lowest *PAr* of the three strata, possibly indicating gene flow from shallow and deep strata to the intermediate depth stratum (Table 2).

**Table 2:** The number of lake trout sampled and genetic diversity across water depth strata

Water depth stratum	*N*	MG	BS	HS	LM	*A*	*A*_R (*i =* 88)_	*H*_O_	*H*_E_	*PA*_R_	*F*_IS_
<50 m	126	83	118	125	118	8.7	8.1	0.57	0.58	0.93	0.02
50–100 m	315	217	308	296	288	10.2	7.7	0.55	0.58	0.79	0.05
>100 m	152	71	121	144	118	8.1	7.4	0.53	0.57	0.94	0.07

Numbers of individuals (*N*) used for microsatellite genotyping (MG), geometric morphometric analyses for body (BS) and head shape (HS), and linear morphometric analyses (LM) are shown along with results for mean number of alleles (*A*), allelic richness (*A*_R_) standardized to the smallest number of alleles per locus (*i*) indicated in parentheses, observed heterozygosity (*H*_O_), expected heterozygosity (*H*_E_), private allelic richness (*PA*_R_), and inbreeding coefficient (*F*_IS_)

### Genetic structure within and among groups

Individual lake trout genotypes showed a lack of clustering in PCOA, and principal coordinate axis 1 (*PC*1 4.2 %) and 2 (*PC*2 3.4 %) had little explanatory power (<8 %) (Additional file 5). Similar to PCOA, Bayesian clustering in STRUCTURE could not distinguish among ecotype, zone, or depth stratum (not shown). However, Bayesian analyses using ‘zone’ and ‘depth stratum’ priors revealed the interaction between zone and depth (see Additional file 6). Individuals captured in the intermediate depth stratum in Zone 3 (see Fig. 1), may be weakly genetically differentiated from all other fish sampled around Isle Royale. However, the group structure was not robust enough for *Q*-values (proportion of ancestry to a given group) to assign individuals to more than one inferred clusters (see Additional file 7). All Jost *D* pairwise comparisons among depths were significant (Table 3). Genetic differentiation estimates for both *Fst* and Jost *D* were largest between the shallow and deep strata, consistent with the hypothesis of a genetic cline with depth.

**Table 3:** Genetic differentiation among lake trout at three water depth strata

	Number	<50 m	50 m–100 m	>100 m
<50 m	66	-	0.030*	0.046*
50–100 m	139	0.005	-	0.035*
>100 m	37	0.012*	0.002	-

*Fst* [68] values calculated in ARLEQUIN and Jost *D* [70] values calculated in R program [57] package ‘DEMEtics’ [69] are below and above the diagonal, respectively. *N* represents sample sizes. Asterisks mark entries with *P*-values that remain significant after correction for multiple tests. Note that only Zones 1 and 2 were included the strata differentiation analysis

Distance matrices between the neutral genetic and the six depth categories (N.B.: six depth categories used for this analysis only) were moderately positively associated (Mantel test: *r*_m_ = 0.45, *P* = 0.047). However, this correlation was significant only after interpolating a single putative outlier (original and adjusted plots and *P*-values shown in Additional file 8). The correlation between genetic and geographic distance (by shortest distance) among 20 samples sites, however, was not significant (Mantel test: *r*_m_ = −0.05, *P* = 0.7).

**Fig. 3:**
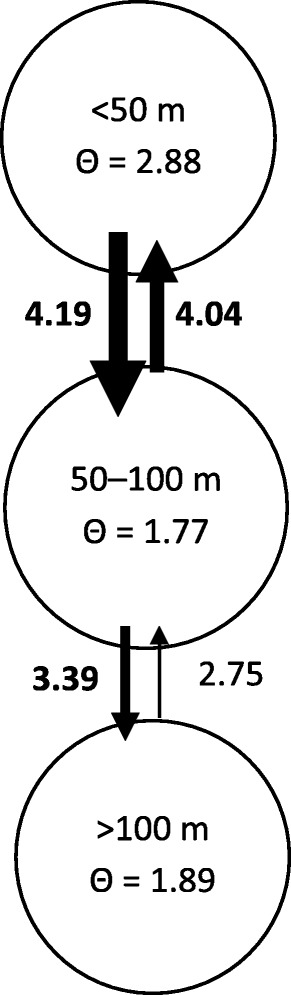
Historical gene flow and effective population sizes (*Ne*) in Isle Royale lake trout. Migration estimates (Ne*m) for historical gene flow greater than 2.5 are shown, as well as the value of theta (*Θ* = 4Ne*μ generations) for each water depth group (as calculated using MIGRATE 3.03 [72, 73]. The *thickness of arrows* corresponds to the relative strength of migration within each analysis

Finally, historical migration rates estimated using the maximum likelihood approach in MIGRATE revealed the direction of gene flow was from shallow to intermediate depth, then intermediate to deep depth (Fig. 3). According to overlap in the 95 % likelihood percentiles (not shown), historical migration estimates appeared to be bidirectional in the upper two depth strata.

### Phenotypic trait variation with water depth

**Fig. 4:**
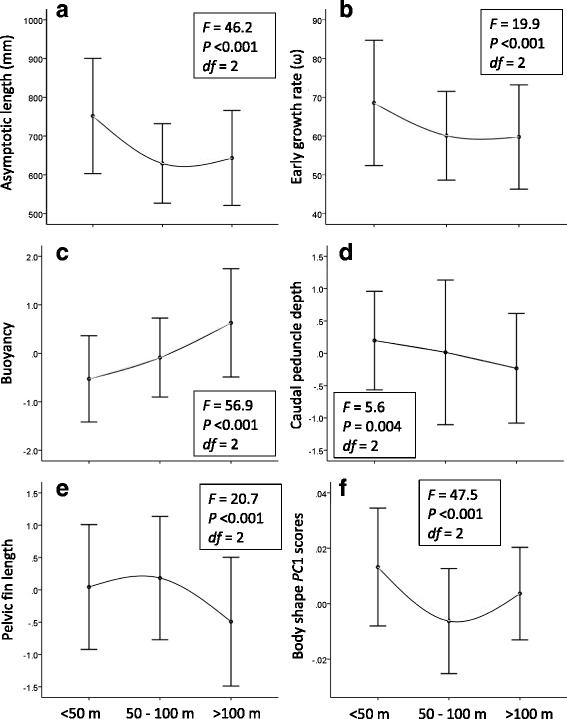
Variation in phenotype with water depth; asymptotic length (**a**), early growth rate (**b**), buoyancy (**c**), caudal peduncle depth (**d**), pelvic fin length (**e**), and body shape *PC*1 (**f**). The studentized residuals from regressions of log_10_ transformed morphological trait measurements against standard length are reported here as size-adjusted variables; asymptotic length, early growth rate, and body shape *PC*1 are raw data. Error bars represent the 95 % confidence interval based on standard deviation of the mean. ANOVA results are reported in each panel (post hoc results available upon request)

Several phenotypic traits were related to depth (Fig. 4). Buoyancy increased clinally with increasing depth, while caudal peduncle depth (and caudal peduncle length, not shown) decreased. Although pelvic fin length and the *PC*1 scores of body shape also decreased with depth, clines were not apparent for these two traits. Similarly, asymptotic length and early growth rate decreased from shallow to deep strata, yet were similar between intermediate and deep strata (Fig. 4). Within-group phenotypic variance (grouped by depth stratum) for pelvic fin length increased significantly with increasing depth, as shown by non-overlapping confidence intervals around the within-group point estimate (Additional file 9). Within-group variance for several other phenotypic traits (e.g., body shape, head shape, caudal peduncle length) significantly decreased with depth. Among life history traits, within-group variance in asymptotic length decreased significantly with depth.

### Relationship between morphology and genotype

**Fig. 5:**
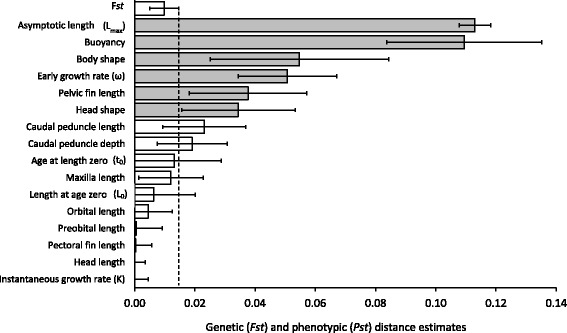
Global phenotypic trait divergence (*Pst*), and analogous measures based on 18 microsatellite DNA loci (*Fst*), for paired-comparisons of lake trout among water depth strata. The *horizontal error bars* indicate 95 % confidence intervals for the *Fst* (*top bar*) and *Pst* (*all other bars*) estimates. Combined effects of natural selection and random drift determine the *Pst* estimates, whereas the *Fst* estimates are determined by drift processes. Lower confidence limits of *Pst* estimates that fall to the left- and right-hand sides of the *dashed vertical line* (upper confidence limit of *Fst*) indicate the effect of drift and selection, respectively, on phenotypic trait divergence. *Horizontal bars* shaded in grey highlight phenotypic traits that putatively exceed neutral expectations based on *Fst*

**Fig. 6:**
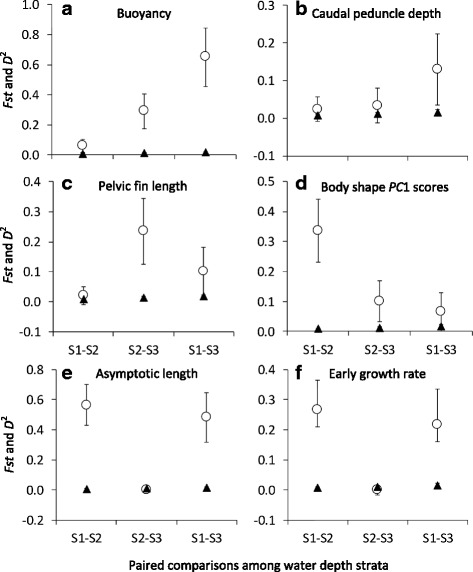
Paired comparisons of phenotypic (*D* ^2^) and genetic (*Fst*) divergence among lake trout in three water depth strata, <50 m (S1), 50–100 m (S2), and >100 m (S3). *Open circles* represent *D* ^2^-values for buoyancy (**a**), caudal peduncle depth (**b**), pelvic fin length (**c**), *PC*1 scores of body shape (**d**), asymptotic length (**e**), and early growth rate (**f**). *Black triangles* represent *Fst*-values calculated in the R package ‘diveRsity’. Error bars indicate 95 % confidence intervals based on bootstrap standard errors. Note that the upper and lower confidence limits around *Fst* were converted to standard error for direct comparison with *Pst*

Six morphological and life history trait divergence estimates (*Pst*-values) among depth strata were significantly higher than corresponding microsatellite-based neutral genetic divergence estimates *Fst*-values: asymptotic length, buoyancy, body and head shape, early growth rate, and pelvic fin length (Fig. 5). Because heritability was set to 1.0, our *Pst* estimates were conservative and prone to false negatives (Type II Errors). Thus, caudal peduncle length and depth, age at length zero, and maxilla length may also be traits that exceed neutral expectations. If the difference between *Pst*-*Fst* values increases with increasing water depth, it may mean that divergent selection increases with water depth, i.e., phenotype becomes more specialized with water depth. Therefore, we conducted paired comparisons of Mahanalobis phenotypic distances, *D* ^2^-values, for buoyancy and caudal peduncle depth increased between depth strata, yet *D* ^2^-values decreased for the *PC*1 metric on body shape (Fig. 6a-d). *D* ^2^-values for life history traits asymptotic length and early growth rate did not vary between shallow-intermediate and shallow-deep strata (Fig. 6e-f)

## Discussion

Despite concern raised from recently documented overlap in morphological [25] and genetic diversity [41] among putative lake trout ecotypes, our results confirmed water depth as an ecological axis of divergence at Isle Royale, Lake Superior that can maintain or promote lake trout diversity. Evidence presented herein supports the hypothesis of genetic and phenotypic divergence along a water depth axis, in which gene flow was partially restricted. This pattern of gene flow suggests that lake trout were not in a state of panmixis. However, high gene flow and lack of strong genetic discontinuities among ecotypes suggest that lake trout genetic diversity, as represented by our collections, was organised along a continuum, rather than among discrete ecotypes, as was historically reported [43, 44, 81]. The exact number of discrete ecotypes at Isle Royale either may be underestimated or possibly cannot be determined using conventional methods. Therefore, we believe the level of lake trout differentiation may have been disrupted from the past and may now have been reset to an early stage of ecotype formation. Reduced diversity may render contemporary lake trout more sensitive to environmental perturbations. Below, we discuss how adaptive differentiation in lake trout ecotypes currently exists along an ecological gradient, their presumed stage of divergence along an ecological speciation continuum, and implications of our findings for species conservation and adaptive diversity in lake trout.

### Water depth as a driver of genetic and phenotypic differentiation

Our findings supported the hypothesis that lake trout genetic and phenotypic variation are divergent along a water depth gradient at Isle Royale, Lake Superior. Despite a lack of significant genetic structure among ecotypes, a consistent pattern in genetic variation among depth strata suggests restricted gene flow by water depth, rather than by ecotype or by geography. Based on our results, water depth appears to be a strong ecological axis of divergence important to maintenance of organization of lake trout genetic and morphological diversity. Vertical distribution of spawning depth often explains neutral genetic group structure (e.g., [21, 82]). Unfortunately, little is known about lake trout spawning areas below 30 m due to logistical difficulties of sampling at great depths [83] (but see [84]), and during inclement weather of late autumn. Lake trout are thought to spawn at depths from 3 to at least 80 m [85], yet most documented egg observations have been at depths <20 m [51, 83, 84, 86, 87]. Variation in local adaptation to water depth we observed, likely depends on selective mechanisms (e.g., survival and reproductive success) on adaptive genes; but also on trait plasticity due to gene expression [4, 8, 88], often tied closely to the environment during development [6]. Therefore, depth at which eggs are spawned and larvae are reared probably influences genetic variation and phenotypic expression in lake trout.

Size-corrected morphological traits, buoyancy, body shape, and pelvic fin length, diverged along the depth axis and appeared to exceed neutral expectations. These traits are likely tied to overall fitness of lake trout and their ability to distribute themselves vertically in the water column. Increased lipid metabolism leading to higher buoyancy is important in deep, cold, dense waters to behaviorally thermoregulate body temperature, and to maintain or change position in the water column [89, 90]. Large pelvic fins function to stabilize body position, and may have a fitness advantage in shallow water considering variable water current speeds, tributary outflows, and periodic storm events [91]. A deep caudal peduncle is critical to life in shallow and fluvial waters, especially for ecotypes that predate on fast moving prey [92–94]. Because the *Pst-Fst* method (see [23]) cannot distinguish between variation from epigenetic-based plasticity and directional selection on genes, the mechanistic basis of lake trout trait evolution remains unclear [21, 22]. Evidence for a degree of genetic accommodation, or hard-wiring, of lipid metabolism has been previously reported (e.g., [35, 38, 39]). On the other hand, buoyancy, body shape, and lipid metabolism are more responsive to environmental stimuli than cranioskeletal traits [95, 96]. Buoyancy in Isle Royale lake trout varied more widely with depth than any other trait we measured, perhaps indicating a higher degree of environmental responsiveness relative to other traits. Although pelvic fin length decreased from shallow to deep strata, measurements were similar between the two shallowest strata. Consequently, pelvic fin length decreased from shallow to deep water for all ecotypes, except for leans (not shown). Possibly, pelvic fin length may be under stronger selection in leans than deep water ecotypes, which show higher levels of plasticity related to pelvic fin length than leans.

Of the life history traits studied here, early growth rate and asymptotic length decreased significantly from shallow to deep strata. These differences in growth and length may be associated with limiting factors related to cold (e.g., nutrient availability) and deep (e.g., possible energetic trade-offs between length and buoyancy) environments. Deep water lake trout tend to mature at smaller sizes and grow slower than shallow water ecotypes [48, 52, 97]. In Flathead Lake, Montana, lake trout life history traits diverged between lake trout caught from shallow and deep depths after the introduction of *Mysis diluviana* [97]; a fatty crustacean that lives mainly below the thermocline [98]. Therefore, the ultimate cause of adaptations to deep water were resource partitioning, while the proximate cause was a shift in spawning and rearing depth that followed the diet shift. The difference between shallow and deep water Lake Superior lake trout life history traits, rather than a gradual cline, could possibly imply a spawning or nursery depth threshold (e.g., thermocline), beyond which environmental variables (e.g., water temperature, dietary protein:lipid ratio) affect eggs and growing larvae differentially [99–101].

Variation in lake trout craniofacial traits (e.g., orbital, head, maxilla length) at Isle Royale did not exceed expectations based on the variation at neutral loci, and phenotypic variance was low within (Additional file 9) and among water depth strata (Fig. 6). We expected Isle Royale lake trout craniofacial features to vary with depth due to presumably different prey assemblages at different depths [102, 103]. Alternatively, apparent neutral evolution of craniofacial traits may suggest stabilizing selection, wherein intermediate variants are favoured among depths, genetic diversity decreases, and the group mean stabilizes on a particular trait. Our results are consistent with studies of Arctic charr *Salvelinus alpinus*, where cranioskeletal genes were expressed differentially when exposed to different food types and were less diverse, and craniofacial traits were less responsive to novel environments than those associated with lipid levels and body shape [3, 95]). However, phenotypic variance in head shape as a whole did exceed neutral expectations when compared to neutral expectations based on genetic divergence in microsatellite loci (as measured by *Fst*). Thus, perhaps prey- or water depth-based selection or phenotypic plasticity has occurred or is still occurring to a degree. Nonetheless, head shape varied less than body shape at Isle Royale. By contrast, at Great Bear Lake (66.0 ° N, 121.0 ° W), head shape varied more than body shape among piscivorous and invertivorous lake trout ecotypes collected from shallow water <30 m [29, 30, 104]. Clearly, lake trout organization is influenced by water depth at Isle Royale. Perhaps the apparent fixation of individual linear craniofacial traits (not head shape as a whole) in Isle Royale lake trout reflect a strong prey resource gradient that originally existed in the lake, and potentially may reduce ability to adapt to exotic introductions or fish community changes [105, 106].

Our results suggest that hard-wiring at adaptive genes in the presence of high gene flow occurred differentially among traits (e.g., craniofacial versus body shape). Similarly, genetically differentiated intraspecific groups vary in the relative proportions of genetic correlations with different traits in three-spine stickleback *Gasterosteus aculeatus* [107–109], whitefish (*Coregonus* sp.; [22]), rainbow trout *Oncorhynchus mykiss* [110], brown trout *Salmo trutta* [111], and Arctic charr *Salvelinus alpinus* [112]. Each trait likely has a different balance of genetic control and environmental responsiveness that varies among lakes, depending on respective selective pressures and their duration and intensity over generations [95]; also see [8, 113, 114].

### Stage of ecological speciation in Lake Superior lake trout

Consistent with a species at an early stage of speciation, we found that four visually distinct lake trout ecotypes could not be distinguished from each other genetically using various population genetic methods for neutral genetic variation. PCOA and Bayesian clustering results on neutral microsatellite loci implied a single panmictic lake trout population (Additional files 5 and 6). However, we found evidence for weak genetic isolation in the form of partially restricted gene flow associated with increasing depth of capture. Increasing phenotypic variation with depth possibly indicated diversifying selection on adaptive genes. Therefore, lake trout ecotypes are likely at an early evolutionary stage characterized by disruptive natural selection on functional phenotypic traits and high gene flow (i.e., between the second and third stages as outlined by Bird et al. [1] and likely reset from a more advanced stage from the past [41]. Lake Superior lake trout are part of a large post-glacial adaptive radiation that spans boreal North America involving at least 20 extant ecotypes in several large (>2000 km^2^) deep cold water lakes [115, 116]. Sufficient generations have passed since the last glacial maximum in the presence of ecological niche diversification (or heterogeneity) to allow parallel development of resource polymorphism in this species in several lakes across North America [27–30, 116]. In Lake Superior, however, gene flow seems high among ecotypes and appears to have increased in recent decades (see [41]), which may have impeded or reversed the historical trajectory of ecological speciation [117]. Although our findings imply that the predominant direction of historical gene flow was from shallow to deep water, evidence of bi-directional gene flow is consistent with phenotypic mixing between the two shallowest depth strata. Considering that low gene flow is expected in the later stages of ecological speciation, lake trout are not likely experiencing strong forward speciation (toward greater divergence) in Lake Superior [118].

### Implications for species conservation and adaptive diversity in lake trout

Lake trout fisheries have targeted the shallow water lean ecotype, whereas humper, siscowet, and redfin typically located farther offshore were fished less. Intensive stocking after the lake trout fishery collapse of the 1950s focussed solely on re-introductions of the lean ecotype during the 1960s through 1990s [36, 119]. Likewise, invasive sea lamprey predation is thought to have had the most impact in shallow water strata [120–122]. The selective fishery and sea lamprey predation may have disproportionately elevated mortality of leans, thereby enhancing fitness of deep water ecotypes and altering the fitness landscape in the lake [21]. Ecological divergence is thought to accelerate when two or more ecological gradients coincide [1, 5, 123]. If lake trout populations were historically divergent on multiple niche axes (e.g., depth and diet simultaneously) and in modern times the number of niche axes has been reduced (e.g., homogenization of diet diversity), then speciation possibly has been impeded, reversed, or reset back to an earlier stage of diversification [124, 125]. At present, whether lake trout diversity in Lake Superior is in the process of collapsing, expanding, or in a stable gene flow-diversification balance cannot be determined. However, based on available information about Lake Superior lake trout, a depth gradient persists and remains evolutionarily important, and ecotypes have at least a partial genetic basis for divergence (see [38]).

Lake trout reproductive boundaries are not completely isolated in Lake Superior, and their evolutionary potential may be especially vulnerable to future changing environmental conditions, and to expansions and reductions in ecological opportunity. Lake trout of Lake Superior appear to have collapsed genetically and morphologically, concurrent with dramatic overall depletions in allelic richness since the 1940s [25, 41]. To discern the processes potentially underlying these losses (e.g., random genetic drift due to population reduction), changes in the selective environment, the nature of gene flow between wild populations and hatchery strains, and adaptive genetic diversity must be better understood. Future genetic analyses of historical collections of lake trout scale samples dating back to before the fishery collapse could shed light on temporal trends in genetic structure and ecological diversity. Such research could permit identification of genetic predictors of human impacts on evolutionary processes in wild populations.

## Conclusions

We provide evidence of reductions in gene flow and divergent natural selection associated with water depth in Lake Superior. These patterns in genetic variation among depth strata persist despite a lack of significant genetic structure when the data set is divided by ecotype. Thus, water depth appears to be a strong ecological axis of divergence important to the maintenance of organization of lake trout genetic and morphological diversity. Lake Superior is the largest freshwater lake in the world, and lake trout have recently lost considerable genetic diversity [41]. Therefore, the results represented in this study are relevant for documenting intraspecific biodiversity for this species. However, it is still unclear as to whether the observed patterns are a result of an early stage of incipient speciation, gene flow-selection equilibrium, or reverse speciation causing formerly divergent ecotypes to collapse into a single gene pool.

## Additional files

Additional file 1: Landmark order and placement for digitizing head and body shape of lake trout. (DOCX 397 kb)
Additional file 2: Lake trout microsatellite primer information. Ta refers to annealing temperature. (DOCX 25 kb)
Additional file 3: Allelic diversity in the combined sample of 371 lake trout from Isle Royale, Lake Superior. Columns indicate the number of individuals genotyped (N), mean number of alleles (A), observed heterozygosity (Ho), expected heterozygosity (He), inbreeding coefficient (Fis) and the *P*-values of Hardy-Weinberg equilibrium (HW). Asterisks mark entries with *P*-values that remain significant after sequential goodness of fit correction for multiple comparisons in ‘SGoF’ 3.8 [58]. (DOCX 22 kb)
Additional file 4: Allelic and genetic diversity statistics for lake trout at Isle Royale, Lake Superior divided by ecotype (A) and sampling zone (B). Columns indicate the number of individuals genotyped (N), mean number of alleles (A), allelic richness (Ar) standardized to the smallest number of alleles per locus (i) indicated in parentheses, observed heterozygosity (Ho), expected heterozygosity (He), private allelic richness (PAr), and inbreeding coefficient (Fis). (DOCX 22 kb)
Additional file 5: First and second principal coordinate (PC) scores (PC1 and PC2) of an individual-based PC analysis on ecotype genotype. Populations were grouped by approximate confidence ellipses for each ecotype (lean = black circles; humper = light grey circles; siscowet = dark grey circles; redfin = red circles). (DOCX 67 kb)
Additional file 6: Bayesian genetic population structure barplots for K = 2 to K = 4 (top) as implemented in program STRUCTURE using 15 loci for Isle Royale lake trout populations divided by three zones and three water depth strata. The light blue, medium blue, and dark blue bands above the plots indicate Zone 1, Zone 2, and Zone 3. Plots of the mean of estimated natural log probably of K [L(K)] (bottom left) and delta K (∆K) to determine number of populations (K) (bottom right) were made using the Evanno in program STRUCTURE HARVESTER Web 0.6.92. (DOCX 158 kb)
Additional file 7: Membership coefficients (Q; computed using STRUCTURE) at K = 2 to K = 4 group clusters for Isle Royale lake trout genotypes divided by from three water depth strata (<50 m, 50–100 m, >100 m) and three zones. The highest Q scores for likelihood of membership for each inferred cluster, QI to QIV, are shaded in grey. (DOCX 22 kb)
Additional file 8: Mantel test results as implemented in GENALEX [62] on matrix correlation (rm) between genetic distance and water depth (a and b), and genetic distance and geographic distance (c). The relationship shown in graph ‘a’ was significant after interpolation of the data point highlighted. The original non-significant relationship and outlier data point are shown in graph ‘b’. (DOCX 93 kb)
Additional file 9: Within-group phenotypic variance based on diagonal of the R matrix model and standard error as calculated in program RMET 5.0 [76–78]. (DOCX 22 kb)

## References

[1] Bird CE, Fernandez-Silva I, Skillings DJ, Toonen RJ (2012). Sympatric speciation in the Post “Modern Synthesis” Era of evolutionary biology. Evol Biol.

[2] Kirk H, Freeland JR (2011). Applications and implications of neutral versus non-neutral markers in molecular ecology. Int J Mol Sci.

[3] Kuettner E, Parsons KJ, Easton AA, Skulason S, Danzmann RG, Ferguson MM (2014). Hidden genetic variation evolves with ecological specialization: the genetic basis of phenotypic plasticity in Arctic charr ecomorphs. Evol Dev.

[4] Skinner MK, Gurerrero-Bosagna C, Haque MM, Nilsson EE, Koop JAH, Knutie SA (2014). Epigenetics and the evolution of Darwin’s finches. Genome Biol Evol.

[5] Wellborn GA, Langerhans RB (2015). Ecological opportunity and the adaptive diversification of lineages. Ecol Evol.

[6] West-Eberhard MJ (2005). Developmental plasticity and the origin of species differences. Proc Natl Acad Sci U S A.

[7] Braendle C, Flatt T (2006). A role for genetic accommodation in evolution?. Bioessays.

[8] Pigliucci M, Murren CJ, Schlichting CD (2006). Phenotypic plasticity and evolution by genetic assimilation. J Exp Biol.

[9] Taylor EB, Boughman JW, Groenenboom M, Sniatynski M, Schluter D, Gow JL (2006). Speciation in reverse: morphological and genetic evidence of the collapse of a three-spined stickleback (*Gasterosteus aculeatus*) species pair. Mol Ecol.

[10] Grant PR, Grant BR (1994). Phenotypic and genetic-effects of hybridization in Darwins finches. Evolution.

[11] Todd TN, Stedman RM (1989). Hybridization of ciscoes (*Coregonus* Spp) in Lake Huron. Can J Zool.

[12] Bhat S, Amundsen P, Knudsen R, Gjelland KO, Fevolden S, Bernatchez L (2014). Speciation reversal in European whitefish (*Coregonus lavaretus* (L.)) caused by competitor invasion. PloS One.

[13] Wilson SD, Keddy PA (1986). Species competitive ability and position along a natural stress disturbance gradient. Ecology.

[14] Richman AD, Price T (1992). Evolution of ecological differences in the Old-World leaf warblers. Nature.

[15] Austin MP, Nicholls AO, Doherty MD, Meyers JA (1994). Determining species response functions to an environmental gradient by means of a beta-function. J Veg Sci.

[16] Schneider CJ, Smith TB, Larison B, Moritz C (1999). A test of alternative models of diversification in tropical rainforests: Ecological gradients vs. rainforest refugia. Proc Natl Acad Sci U S A.

[17] Seehausen O, van Alphen JJM, Witte F (1997). Cichlid fish diversity threatened by eutrophication that curbs sexual selection. Science.

[18] Magalhaes IS, Seehausen O (2010). Genetics of male nuptial colour divergence between sympatric sister species of a Lake Victoria cichlid fish. J Evol Biol.

[19] Turgeon J, Reid SM, Bourret A, Pratt TC, Reist JD, Muir AM (2016). Morphological and genetic variation in cisco (*Coregonus artedi*) and shortjaw cisco (*C. zenithicus*): multiple origins of shortjaw cisco in inland lakes require a lake-specific conservation approach. Conserv Genet.

[20] Helland IP, Harrod C, Freyhof J, Mehner T (2008). Co-existence of a pair of pelagic planktivorous coregonid fishes. Evol Ecol Res.

[21] Vonlanthen P, Roy D, Hudson AG, Largiader CR, Bittner D, Seehausen O (2009). Divergence along a steep ecological gradient in lake whitefish (*Coregonus* sp.). J Evol Biol.

[22] Praebel K, Knudsen R, Siwertsson A, Karhunen M, Kahilainen KK, Ovaskainen O (2013). Ecological speciation in postglacial European whitefish: rapid adaptive radiations into the littoral, pelagic, and profundal lake habitats. Ecol Evol.

[23] Leinonen T, McCairns RJS, O’Hara RB, Merila J (2013). *Q*_ST_-*F*_ST_ comparisons: evolutionary and ecological insights from genomic heterogeneity. Nat Rev Genet.

[24] Krueger CC, Ihssen PE (1995). Review of genetics of lake trout in the great lakes: History, molecular genetics, physiology, strain comparisons, and restoration management. J Great Lakes Res.

[25] Muir AM, Bronte CR, Zimmerman MS, Quinlan HR, Glase JD, Krueger CC (2014). Ecomorphological diversity of lake trout at Isle Royale, Lake Superior. Trans Am Fish Soc.

[26] Harvey CJ, Schram ST, Kitchell JF (2003). Trophic relationships among lean and siscowet lake trout in Lake Superior. Trans Am Fish Soc.

[27] Zimmerman MS, Krueger CC, Eshenroder RL (2006). Phenotypic diversity of lake trout in Great Slave Lake: Differences in morphology, buoyancy, and habitat depth. Trans Am Fish Soc.

[28] Zimmerman MS, Krueger CC, Eshenroder RL (2007). Morphological and ecological differences between shallow- and deep-water lake trout in Lake Mistassini, Quebec. J Great Lakes Res.

[29] Chavarie L, Howland KL, Tonn WM (2013). Sympatric polymorphism in lake trout: The coexistence of multiple shallow-water morphotypes in Great Bear Lake. Trans Am Fish Soc.

[30] Chavarie L, Howland K, Harris L, Tonn W (2015). Polymorphism in lake trout in Great Bear Lake: intra-lake morphological diversification at two spatial scales. Biol J Linn Soc.

[31] Behnke RJ (1972). Systematics of salmonid fishes of recently glaciated lakes. J Fish Res Board Can.

[32] Ray BA, Hrabik TR, Ebener MP, Gorman OT, Schreiner DR, Schram ST (2007). Diet and prey selection by Lake Superior lake trout during spring, 1986–2001. J Great Lakes Res.

[33] Bronte C, Ebener M, Schreiner D, DeVault D, Petzold M, Jensen D (2003). Fish community change in Lake Superior, 1970–2000. Can J Fish Aquat Sci.

[34] Rahrer JF (1965). Age growth maturity and fecundity of humper lake trout Isle Royale Lake Superior. Trans Am Fish Soc.

[35] Eschmeyer PH, Phillips AM (1965). Fat content of flesh of siscowets and lake trout from Lake Superior. Trans Am Fish Soc.

[36] Hansen MJ, Peck JW, Schorfhaar RG, Selgeby JH, Schreiner DR, Schram ST (1995). Lake trout (*Salvelinus namaycush*) populations in Lake Superior and their restoration in 1959–1993. J Great Lakes Res.

[37] Ihssen P, Tait JS (1974). Genetic differences in retention of swimbladder gas between two populations of lake trout (*Salvelinus namaycush*). J Fish Res Board Can.

[38] Goetz F, Rosauer D, Sitar S, Goetz G, Simchick C, Roberts S (2010). A genetic basis for the phenotypic differentiation between siscowet and lean lake trout (*Salvelinus namaycush*). Mol Ecol.

[39] Goetz F, Sitar S, Rosauer D, Swanson P, Bronte CR, Dickey J (2011). The reproductive biology of siscowet and lean lake trout in southern Lake Superior. Trans Am Fish Soc.

[40] Bronte CR, Sitar SP (2008). Harvest and relative abundance of siscowet lake trout in Michigan waters of Lake Superior, 1929–1961. Trans Am Fish Soc.

[41] Baillie, SM, Muir, AM, Krueger, CC, Scribner, K, Bentzen, P. Loss of genetic diversity and reduction of genetic distance among lake trout *Salvelinus namaycush* ecomorphs, Lake Superior 1959 to 2013. J Great Lakes Res. doi: 10.1016/j.jglr.2016.02.001

[42] Taylor EB (2010). Changes in taxonomy and species distributions and their influence on estimates of faunal homogenization and differentiation in freshwater fishes. Divers Distrib.

[43] Guinand B, Scribner KT, Page KS, Burnham-Curtis MK (2003). Genetic variation over space and time: analyses of extinct and remnant lake trout populations in the Upper Great Lakes. Proc R Soc London, Ser B.

[44] Guinand B, Page KS, Burnham-Curtis MK, Scribner KT (2012). Genetic signatures of historical bottlenecks in sympatric lake trout (*Salvelinus namaycush*) morphotypes in Lake Superior. Environ Biol Fishes.

[45] Zelditch ML, Swiderski DL, Sheets DH, Fink WL (2004). Geometric morphometrics for biologists: a primer.

[46] Zimmerman MS, Schmidt SN, Krueger CC, Vander Zanden MJ, Eshenroder RL (2009). Ontogenetic niche shifts and resource partitioning of lake trout morphotypes. Can J Fish Aquat Sci.

[47] Fraley C, Raftery AE (2009). MCLUST version 3 for R: normal mixuture modeling and model-based clustering.

[48] Hansen. MJ, Nate, NA, Muir, AM, Bronte, CR, Zimmerman, MS, Krueger, CC. Life history variation among four lake trout morphs at Isle Royale, Lake Superior. J Great Lakes Res. doi:10.1016/j.jglr.2015.12.011.

[49] Campana SE (1990). How reliable are growth back-calculations based on otoliths. Can J Fish Aquat Sci.

[50] Gallucci VF, Quinn TJ (1979). Reparameterizing, fitting, and testing a simple growth-model. Trans Am Fish Soc.

[51] Bronte CR, Selgeby JH, Saylor JH, Miller GS, Foster NR (1995). Hatching, dispersal, and bathymetric distribution of age-0 wild lake trout at the Gull Island shoal complex, Lake Superior. J Great Lakes Res.

[52] Hansen MJ, Nate NA, Krueger CC, Zimmerman MS, Kruckman HG, Taylor WW (2012). Age, Growth, Survival, and Maturity of Lake Trout Morphotypes in Lake Mistassini, Quebec. Trans Am Fish Soc.

[53] Elphinstone MS, Hinten GN, Anderson MJ, Nock CJ (2003). An inexpensive and high-throughput procedure to extract and purify total genomic DNA for population studies. Mol Ecol Notes.

[54] Van Oosterhout C, Hutchinson W, Wills D, Shipley P (2004). MICRO-CHECKER: software for identifying and correcting genotyping errors in microsatellite data. Mol Ecol Notes.

[55] Coombs JA, Letcher BH, Nislow KH (2008). CREATE: a software to create input files from diploid genotypic data for 52 genetic software programs. Mol Ecol Resour.

[56] Excoffier L, Lischer HEL (2010). Arlequin suite ver 3.5: a new series of programs to perform population genetics analyses under Linux and Windows. Mol Ecol Resour.

[57] R Core Team. R: A language and environment for statistical computing. Vienna: R Foundation for Statistical Computing. 2015. https://www.r-project.org.

[58] Carvajal-Rodriguez A, de Una-Alvarez J (2011). Assessing significance in high-throughput experiments by sequential goodness of fit and q-value estimation. PloS One.

[59] Goudet J (1995). FSTAT (Version 1.2): A computer program to calculate F-statistics. J Hered.

[60] Kalinowski S (2004). Counting alleles with rarefaction: Private alleles and hierarchical sampling designs. Conserv Genet.

[61] Wright S (1921). Systems of mating. II. The effects of inbreeding on the genetic composition of a population. Genetics.

[62] Peakall R, Smouse PE (2012). GenAlEx 6.5: genetic analysis in Excel. Population genetic software for teaching and research-an update. Bioinformatics.

[63] Pritchard J, Stephens M, Donnelly P (2000). Inference of population structure using multilocus genotype data. Genetics.

[64] Falush D, Stephens M, Pritchard JK (2007). Inference of population structure using multilocus genotype data: dominant markers and null alleles. Mol Ecol Notes.

[65] Hubisz MJ, Falush D, Stephens M, Pritchard JK (2009). Inferring weak population structure with the assistance of sample group information. Mol Ecol Resour.

[66] Evanno G, Regnaut S, Goudet J (2005). Detecting the number of clusters of individuals using the software STRUCTURE: a simulation study. Mol Ecol.

[67] Earl DA, von Holdt BM (2012). STRUCTURE HARVESTER: a website and program for visualizing STRUCTURE output and implementing the Evanno method. Conserv Genet Resour.

[68] Wright S (1965). The Interpretation of Population-Structure by F-Statistics with Special Regard to Systems of Mating. Evolution.

[69] Gerlach G, Jueterbock A, Kraemer P, Deppermann J, Harmand P (2010). Calculations of population differentiation based on *G*ST and *D*: forget *G*ST but not all of statistics!. Mol Ecol.

[70] Jost L (2008). G(ST) and its relatives do not measure differentiation. Mol Ecol.

[71] Manly BFJ (1997). A method for the estimation of parameters for natural stage-structured populations. Res Popul Ecol.

[72] Beerli P, Felsenstein J (2001). Maximum likelihood estimation of a migration matrix and effective population sizes in n subpopulations by using a coalescent approach. Proc Natl Acad Sci U S A.

[73] Beerli P, Palczewski M (2010). Unified framework to evaluate panmixia and migration direction among multiple sampling locations. Genetics.

[74] Weir BS, Cockerham CC (1984). Estimating F-statistics for the analysis of population-structure. Evolution.

[75] Spitze K (1993). Population structure in *Daphnia obtusa* - quantitative genetic and allozymic variation. Genetics.

[76] Relethford JH, Blangero J (1990). Detection of differential gene flow from patterns of quantitative variation. Hum Biol.

[77] Relethford JH, Crawford MH, Blangero J (1997). Genetic drift and gene flow in post-famine Ireland. Hum Biol.

[78] Relethford JH (2003). The human species: an introduction to biological anthropology.

[79] Keenan K, McGinnity P, Cross TF, Crozier WW, Prodoehl PA (2013). diveRsity: An R package for the estimation and exploration of population genetics parameters and their associated errors. Methods Ecol Evol.

[80] Seehausen O (2004). Hybridization and adaptive radiation. Trends Ecol Evol.

[81] Page K, Scribner K, Burnham-Curtis M (2004). Genetic diversity of wild and hatchery lake trout populations: Relevance for management and restoration in the Great Lakes. Trans Am Fish Soc.

[82] Florin A, Hoglund J (2008). Population structure of flounder (*Platichthys flesus*) in the Baltic Sea: differences among demersal and pelagic spawners. Heredity.

[83] Marsden JE, Janssen J (1997). Evidence of lake trout spawning on a deep reef in Lake Michigan using an ROV-based egg collector. J Great Lakes Res.

[84] Janssen J, Jude DJ, Edsall TA, Paddock RW, Wattrus N, Toneys M (2006). Evidence of lake trout reproduction at Lake Michigan’s Mid-Lake Reef Complex. J Great Lakes Res.

[85] Marsden JE, Casselman JM, Edsall TA, Elliott RF, Fitzsimons JD, Horns WH (1995). Lake trout spawning habitat in the Great Lakes - A review of current knowledge. J Great Lakes Res.

[86] Janssen J, Marsden JE, Bronte CR, Jude DJ, Sitar SP, Goetz FW (2007). Challenges to deep-water reproduction by lake trout: Pertinence to restoration in Lake Michigan. J Great Lakes Res.

[87] Maclean JA, Evans DO, Martin NV, Desjardine RL (1981). Survival, growth, spawning distribution, and movements of introduced and native lake trout (*Salvelinus-namaycush*) in two inland Ontario lakes. Can J Fish Aquat Sci.

[88] Renaut S, Nolte AW, Bernatchez L (2009). Gene expression divergence and hybrid misexpression between lake whitefish species pairs (*Coregonus* spp. Salmonidae). Mol Biol Evol.

[89] Phleger CF (1998). Buoyancy in marine fishes: Direct and indirect role of lipids. Am Zool.

[90] Henderson BA, Anderson DM (2002). Phenotypic differences in buoyancy and energetics of lean and siscowet lake charr in Lake Superior. Environ Biol Fishes.

[91] Standen EM (2008). Pelvic fin locomotor function in fishes: three-dimensional kinematics in rainbow trout (*Oncorhynchus mykiss*). J Exp Biol.

[92] Lauder GV, Drucker EG (2004). Morphology and experimental hydrodynamics of fish fin control surfaces. IEEE J Ocean Eng.

[93] Hall B (2007). Fins into Limbs: Evolution, Development, and Transformation University of Chicago Press.

[94] Helfman G, Collette BB, Facey DE, and Bowen BW. Functional morphology of locomotion and feeding. Chapter 8. In: The Diversity of Fishes: Biology. Hoboken, New Jersey: John Wiley & Sons; 2009. pp. 101–116.

[95] Kuettner E, Parsons KJ, Robinson BW, Skulason S, Danzmann RG, Ferguson MM (2013). Effects of population, family, and diet on craniofacial morphology of Icelandic Arctic charr (*Salvelinus alpinus*). Biol J Linn Soc.

[96] Parsons KJ, Marquez E, Cooper WJ, Albertson RC (2010). The genetic basis of modularity in the African cichlid mandible. Integr Comp Biol.

[97] Stafford CP, McPhee MV, Eby LA, Allendorf FW (2014). Introduced lake trout exhibit life history and morphological divergence with depth. Can J Fish Aquat Sci.

[98] Audzijonyte A, Vainola R (2005). Diversity and distributions of circumpolar fresh- and brackish-water *Mysis* (Crustacea: Mysida): descriptions of *M. relicta* Loven, 1862, *M. salemaai n.* sp., *M. segerstralei n.* sp and *M. diluviana n.* sp., based on molecular and morphological characters. Hydrobiologia.

[99] Horns WH (1985). Differences in early development among lake trout (Salvelinus namaycush) Populations. Can J Fish Aquat Sci.

[100] Atse CB, Audet C, de la Noue J (2002). Effects of temperature and salinity on the reproductive success of Arctic charr, *Salvelinus alpinus* (L.): egg composition, milt characteristics and fry survival. Aquac Res.

[101] Bogevik AS, Henderson RJ, Mundheim H, Olsen RE, Tocher DR (2011). The effect of temperature and dietary fat level on tissue lipid composition in Atlantic salmon (*Salmo salar*) fed wax ester-rich oil from *Calanus finmarchicus*. Aquacult Nutr.

[102] Sierszen ME, Peterson GS, Scharold JV (2006). Depth-specific patterns in benthic-planktonic food web relationships in Lake Superior. Can J Fish Aquat Sci.

[103] Sierszen ME, Hrabik TR, Stockwell JD, Cotter AM, Hoffman JC, Yule DL (2014). Depth gradients in food-web processes linking habitats in large lakes: Lake Superior as an exemplar ecosystem. Freshw Biol.

[104] Harris LN, Chavarie L, Bajno R, Howland KL, Wiley SH, Tonn WM (2015). Evolution and origin of sympatric shallow-water morphotypes of Lake Trout, *Salvelinus namaycush*, in Canada’s Great Bear Lake. Heredity.

[105] Christie WJ (1974). Changes in fish species composition of Great Lakes. J Fish Res Board Can.

[106] Mills EL, Leach JH, Carlton JT, Secor CL (1993). Exotic species in the Great-Lakes - a history of biotic crises and anthropogenic introductions. J Great Lakes Res.

[107] Lucek K, Sivasundar A, Seehausen O (2014). Disentangling the role of phenotypic plasticity and genetic divergence in contemporary ecotype formation during a biological invasion. Evolution.

[108] Leinonen T, McCairns RJS, Herczeg G, Merila J (2012). Multiple evolutionary pathways to decreased lateral plate coverage in freshwater threespine sticklebacks. Evolution.

[109] Berner D, Kaeuffer R, Grandchamp A, Raeymaekers JAM, Raesaenen K, Hendry AP (2011). Quantitative genetic inheritance of morphological divergence in a lake-stream stickleback ecotype pair: implications for reproductive isolation. J Evol Biol.

[110] Keeley ER, Parkinson EA, Taylor EB (2007). The origins of ecotypic variation of rainbow trout: a test of environmental vs. genetically based differences in morphology. J Evol Biol.

[111] Meier K, Hansen MM, Bekkevold D, Skaala O, Mensberg K-D (2011). An assessment of the spatial scale of local adaptation in brown trout *(Salmo trutta* L.): footprints of selection at microsatellite DNA loci. Heredity.

[112] Kapralova KH, Franzdottir SR, Jonsson H, Snorrason SS, Jonsson ZO (2014). Patterns of miRNA expression in arctic charr development. PloS One.

[113] Jablonka E (2013). Epigenetic inheritance and plasticity: The responsive germline. Prog Biophys Mol Biol.

[114] Robinson BW (2013). Evolution of growth by genetic accommodation in Icelandic freshwater stickleback. Proc R Soc London, Ser B.

[115] Lawrie AH, Rahrer JF (1972). Lake-Superior - Effects of exploitation and introductions on salmonid community. J Fish Res Board Can.

[116] Muir AM, Hansen MJ, Bronte CR, Krueger CC. If Arctic charr *Salvelinus alpinus* is ‘the most diverse vertebrate’, what is the lake charr *Salvelinus namaycush*? Fish Fish. 2015; doi: 10.1111/faf.12114

[117] Bierne N, Gagnaire P, David P (2013). The geography of introgression in a patchy environment and the thorn in the side of ecological speciation. Curr Zool.

[118] Hendry AP (2009). Ecological speciation! Or the lack thereof?. Can J Fish Aquat Sci.

[119] Krueger CC, Ebener M, Gunn JM, Stedman RJ, Ryder RA (2004). Rehabilitation of lake trout in the Great Lakes: past lessons and future challenges. Boreal Shield watersheds: Lake trout ecosystems in a changing environment.

[120] Kitchell JF (1990). The scope for mortality caused by sea lamprey. Trans Am Fish Soc.

[121] Jorgensen JC, Kitchell JF (2005). Growth potential and host mortality of the parasitic phase of the sea lamprey (*Petromyzon marinus*) in Lake Superior. Can J Fish Aquat Sci.

[122] Moody EK, Weidel BC, Ahrenstorff TD, Mattes WP, Kitchell JF (2011). Evaluating the growth potential of sea lampreys (*Petromyzon marinus*) feeding on siscowet lake trout (*Salvelinus namaycush*) in Lake Superior. J Great Lakes Res.

[123] Seehausen O (2006). Conservation: Losing biodiversity by reverse speciation. Curr Biol.

[124] Nosil P, Harmon LJ, Seehausen O (2009). Ecological explanations for (incomplete) speciation. Trends Ecol Evol.

[125] Ingram T, Hudson AG, Vonlanthen P, Seehausen O (2012). Does water depth or diet divergence predict progress towards ecological speciation in whitefish radiations?. Evol Ecol Res.

