# Supplementary material for: Genetic and phenotypic variation along an ecological gradient in lake trout Salvelinus namaycush
Source: BMC Evol Biol. 2016 Oct 19;16:219. doi: 10.1186/s12862-016-0788-8 (PMC5069848; doi:10.1186/s12862-016-0788-8)
Supplement: Additional file 1: — Landmark order and placement for digitizing head and body shape of lake trout. (DOCX 397 kb) [file 12862_2016_788_MOESM1_ESM.docx]

**
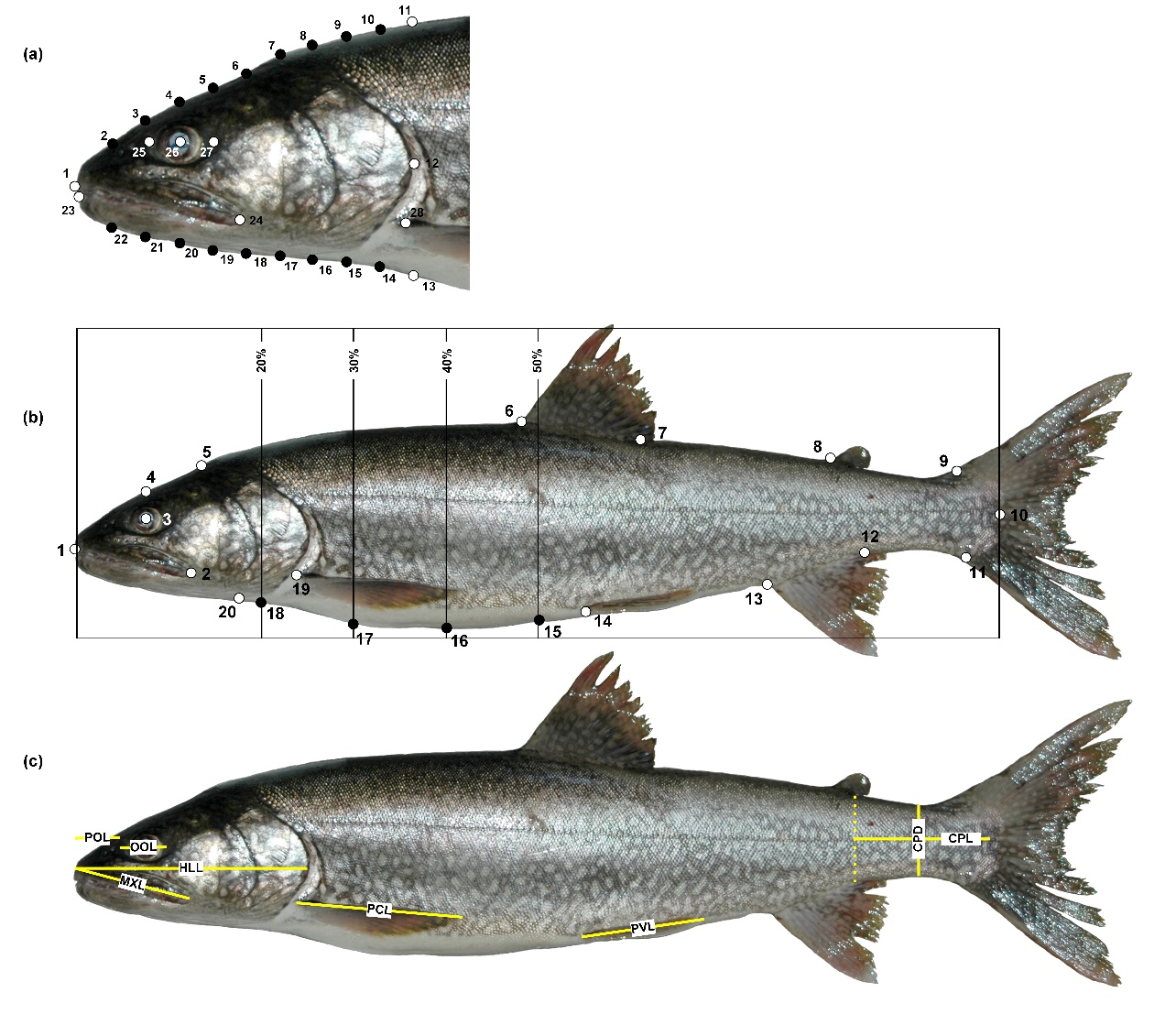
Additional file 1**. Landmark order and placement for digitizing head and body shape of lake trout.

(a) Semi-landmarks (black dots) were placed at 10 evenly spaced partitions between the anterior tip of the snout and the posterior edge of the opercle. Homologous landmarks (white dots) indicate the anterior terminus of upper (1) and lower jaw (23); anterior (25), center (26), and posterior (27) of the orbit; posterior terminus of maxilla (24); pectoral fin insertion (28); and the posterior of the opercle (12). Landmarks 1, 11, 13, and 23 are the anchors between which the semi-landmarks slide. (b) Semi-landmarks (black dots) were placed at 0.20, 0.30, 0.40, and 0.50×standard length (SL) to quantify body depth. Homologous landmarks (white dots) indicated the anterior terminus of upper jaw (1), posterior tip of maxilla (2), center of eye (3), top of cranium at midpoint of eye (4), posterior of neurocranium (5), anterior insertion of dorsal fin (6), posterior insertion of dorsal fin (7), anterior insertion of adipose fin (8), dorsal insertion of caudal fin (9), midpoint of hypural plate (10), ventral insertion of caudal fin (11), posterior insertion of anal fin (12), anterior insertion of anal fin (13), anterior insertion of pelvic fin (14), dorsal insertion of pectoral fin (19), and isthmus of branchiostegal membrane (20; c.f., Zimmerman et al. [46]. (c) Eight linear phenotypic characteristics were measured on each specimen as follows: (1) caudal peduncle depth (CPD): least vertical depth of the caudal peduncle; (2) caudal peduncle length (CPL): distance along the horizontal axis of the body between the posterior of the anal fin and the caudal flexure; (3) head length (HLL): distance from the tip of the premaxilla to the posterior margin of the opercle; (4) maxilla length (MXL): anterior point of premaxillae to posterior end of the maxilla; (5) orbital length (OOL): distance between anterior and posterior fleshy margins of the orbit; (6) pectoral fin length (PCL): measured from the insertion of outermost ray to farthest tip of fin; (7) pelvic fin length (PVL): measured from the insertion of outermost ray to farthest tip of fin; and (8) preorbital length (POL): tip of the premaxilla to the anterior fleshy margin of the orbit. [Figure and caption reproduced directly from Muir et al. [25] with permission.]
