# Supplementary material for: Genetic and phenotypic variation along an ecological gradient in lake trout Salvelinus namaycush
Source: BMC Evol Biol. 2016 Oct 19;16:219. doi: 10.1186/s12862-016-0788-8 (PMC5069848; doi:10.1186/s12862-016-0788-8)
Supplement: Additional file 2: — Lake trout microsatellite primer information. Ta refers to annealing temperature. (DOCX 25 kb) [file 12862_2016_788_MOESM2_ESM.docx]

**Additional file 2**. Lake trout microsatellite primer information. Ta refers to annealing temperature.

| Primer | Primer Sequence (5' - 3') | Repeat Motif | Ta | GenBank Accession No. | Reference |
| --- | --- | --- | --- | --- | --- |
| Ssa85 | F: AGGTGGGTCCTCCAAGCTAC | (GT)_14_ | 60 | NA | O'Reilly et al. 1996 |
|  | R: ACCCGCTCCTCACTTAATC |  |  |  |  |
| Sco215 | F: GAGAGAGAGAGATGGGTGACA | (GAAA)_6_ (GA)_6_ (GGGA)_1_ (GA)_13_ | 60 | AY88884 | Dehaan and Ardren 2005 |
|  | R: ATCCACAAAACAAGATTGCTA |  |  |  |  |
| Sco202 | F: TTGGTTCCTTCCCCTTAGC | (CTAT)_10_ | 60 | AY88871 | Dehaan and Ardren 2005 |
|  | R: GCTGAAATAGCCGAATCCA |  |  |  |  |
| Ogo1A | F: GATCTGGGCCTAAGGGAAAC | (GTCT)_26_ | 60 | AF007827 | Olsen et al. 1998 |
|  | R: ACTAGCGGTTGGAGAACCC |  |  |  |  |
| Snamsu06 | F: GCTGGTGAGGGAGAGATGAC | (CA)_12_ (GACA)_22_ | 60 | EU331433 | Rollins et al. 2009 |
|  | R: CAGCCATGAGAATGGGATTT |  |  |  |  |
| Snamsu12 | F: ATTTTCCACATGCTGCGTCT | (GTCT)_27_ | 60 | EU331437 | Rollins et al. 2009 |
|  | R: TGAAATAGCTTGGAGCAGTAGC |  |  |  |  |
| Sfo334 | F: GGATTAACAGAAGGTTACTG | (TG)_58_ G_4_(TG)_27_ (CGTG)_8_ | 57 | NA | Perry et al. 2005 |
|  | R: CTTCGTATTCTTCATTGTGC |  |  |  |  |
| Otsg253b | F: GAGCAGGCCGAGCAGGTGTCT | (GACA)_10_ (GATA)_14_ | 57 | AF393193 | Williamson et al. 2002 |
|  | R: AATTGGGTCATTAAGGCTCTGTGG |  |  |  |  |
| Snamsu02 | F: GCCCTTCATTGAGGAACAGA | (GTCT)_2_ GTCAAT (GT)_5_ (GTCT)_11_ GT(CCGT)_15_ | 57 | EU331428 | Rollins et al. 2009 |
|  | R: CTCACACACACGCACAACAA |  |  |  |  |
| Sco102 | F: CCATCTCTTCTTACCCTCCTC | CATC | 57 | NA | Sewall Young (unpublished) |
|  | R: CCAAAAAGCAGTTGATAGACC |  |  |  |  |
| Sco107 | F: TAGGCTTGTCAGCAGTGAG | TAGA | 57 | NA | Sewall Young (unpublished) |
|  | R: CCGAGTTTCAGAGGATGTC |  |  |  |  |
| Otsg83b | F: TAGCCCTGCACTAAAATACAGTTC | (TGTC)_7_ N_51_(TATC)_34_ | 52 | AF393189 | Williamson et al. 2002 |
|  | R: CATTAATCTAGGCTTGTCAGCAGT |  |  |  |  |
| OneU9 | F: CTCTCTTTGGCTCGGGGAATGTT | (CA)_54_ | 60 |  | Scribner et al. 1996 |
|  | R: GCATGTTCTGACAGCCTACAGCT |  |  |  |  |
| SalD39 | F: GGGGAGTCTGTGTTAAGTTGG | (GT)_3_(AC)_23_ | 55 | AF537310 | McGowan et al. 2004 |
|  | R: TGAATGGACGTTCCTCTGAC |  |  |  |  |
| Sazim | F: AACTTCAAGATATATGATGT |  | 44 | NA | Englbrecht 2000 |
|  | R: ATTCGTTTAGTCTGAGAA |  |  |  |  |
| Sco19 | F: CTT GAA ATT AGT TAA ACA GC |  | 55 | NA | Taylor et al. 2001 |
|  | R: CCA AAC TAC CCA ATA ATC |  |  |  |  |
| Sfo12 | F: GGT TTT GAA GAG TGA CAG |  | 52 | NA | Angers et al. 1995 |
|  | R: CCC GTT TCA CAA TCA GAG |  |  |  |  |
| SfoB52 | F: GCA CAC GAA ACC AGT ATA TTT | (GCGT)_12_ | 60 | AY168186 | King et al. 2013 |
|  | R: TTG TCT TGG TGA TTT CAG AGC |  |  |  |  |
| SfoC24 | F: GCT ACT GTT GGA TTT CAT CTC AG | (GAT)_10_ | 63 | AY168187 | King et al. 2012 |
|  | R: ATC ACA GAG ATG GGG TGA TG |  |  |  |  |
