# Supplementary material for: Genetic and phenotypic variation along an ecological gradient in lake trout Salvelinus namaycush
Source: BMC Evol Biol. 2016 Oct 19;16:219. doi: 10.1186/s12862-016-0788-8 (PMC5069848; doi:10.1186/s12862-016-0788-8)
Supplement: Additional file 3: — Allelic diversity in the combined sample of 371 lake trout from Isle Royale, Lake Superior. Columns indicate the number of individuals genotyped (N), mean number of alleles (A), observed heterozygosity (Ho), expected heterozygosity (He), inbreeding coefficient (Fis) and the P-values of Hardy-Weinberg equilibrium (HW). Asterisks mark entries with P-values that remain significant after sequential goodness of fit correction for multiple comparisons in ‘SGoF’ 3.8 [58]. (DOCX 22 kb) [file 12862_2016_788_MOESM3_ESM.docx]

**Additional file 3.** Allelic diversity in the combined sample of 371 lake trout from Isle Royale, Lake Superior. Columns indicate the number of individuals genotyped (*N*), mean number of alleles (*A*), observed heterozygosity (*Ho*), expected heterozygosity (*He*), inbreeding coefficient (*Fis*) and the *P*-values of Hardy-Weinberg equilibrium (HW). Asterisks mark entries with *P*-values that remain significant after sequential goodness of fit correction for multiple comparisons in SGOF 3.8 [58].

| locus | *N* | *A* | *Ho* | *He* | *Fis* | HW |
| --- | --- | --- | --- | --- | --- | --- |
| Ogo1 | 306 | 3 | 0.51 | 0.51 | -0.01 | 0.50 |
| OneU9 | 316 | 6 | 0.05 | 0.05 | 0.04 | 0.03* |
| Otsg83b | 331 | 13 | 0.45 | 0.48 | 0.06 | <0.01* |
| Sal-D39 | 325 | 4 | 0.04 | 0.04 | 0.07 | 0.17 |
| Sazim | 268 | 26 | 0.72 | 0.78 | 0.07 | 0.49 |
| Sco102 | 357 | 6 | 0.23 | 0.21 | -0.07 | 0.75 |
| Sco107 | 341 | 11 | 0.50 | 0.50 | 0.00 | 0.07 |
| Sco19 | 347 | 12 | 0.62 | 0.70 | 0.12 | 0.16 |
| Sco202 | 290 | 14 | 0.81 | 0.87 | 0.06 | 0.05 |
| Sco215 | 326 | 8 | 0.72 | 0.71 | -0.01 | 0.04* |
| Sfo12 | 348 | 5 | 0.26 | 0.25 | -0.02 | 0.05 |
| Sfo334 | 263 | 25 | 0.60 | 0.89 | 0.33 | <0.01* |
| SfoB52 | 275 | 6 | 0.57 | 0.60 | 0.05 | 0.04* |
| SfoC24 | 250 | 3 | 0.66 | 0.60 | -0.10 | 0.19 |
| Snamsu02 | 295 | 22 | 0.79 | 0.86 | 0.08 | 0.23 |
| Snamsu06 | 300 | 25 | 0.87 | 0.91 | 0.05 | <0.01* |
| Snamsu12 | 309 | 15 | 0.90 | 0.89 | -0.01 | 0.48 |
| Ssa85 | 320 | 4 | 0.62 | 0.58 | -0.07 | 0.02* |
