# Supplementary material for: Genetic and phenotypic variation along an ecological gradient in lake trout Salvelinus namaycush
Source: BMC Evol Biol. 2016 Oct 19;16:219. doi: 10.1186/s12862-016-0788-8 (PMC5069848; doi:10.1186/s12862-016-0788-8)
Supplement: Additional file 4: — Allelic and genetic diversity statistics for lake trout at Isle Royale, Lake Superior divided by ecotype (A) and sampling zone (B). Columns indicate the number of individuals genotyped (N), mean number of alleles (A), allelic richness (Ar) standardized to the smallest number of alleles per locus (i) indicated in parentheses, observed heterozygosity (Ho), expected heterozygosity (He), private allelic richness (PAr), and inbreeding coefficient (Fis). (DOCX 22 kb) [file 12862_2016_788_MOESM4_ESM.docx]

**Additional file 4.** Allelic and genetic diversity statistics for lake trout at Isle Royale, Lake Superior divided by ecotype (A) and sampling zone (B). Columns indicate the number of individuals genotyped (*N*), mean number of alleles (*A*), allelic richness (*Ar*) standardized to the smallest number of alleles per locus (*i*) indicated in parentheses, observed heterozygosity (*Ho*), expected heterozygosity (*He*), private allelic richness (*PAr*), and inbreeding coefficient (*Fis*).

| A. | *N* | *A* | *Ar*  _(_*_i =_* _51)_ | *Ho* | *He* | *PAr* | *Fis* |
| --- | --- | --- | --- | --- | --- | --- | --- |
| Lean | 105 | 9.2 | 8.4 | 0.56 | 0.59 | 0.82 | 0.04 |
| Humper | 52 | 7.2 | 7.2 | 0.54 | 0.56 | 0.28 | 0.05 |
| Siscowet | 163 | 9.9 | 8.1 | 0.54 | 0.58 | 0.71 | 0.06 |
| Redfin | 51 | 7.8 | 7.8 | 0.56 | 0.58 | 0.43 | 0.03 |

| B. | *N* | *A* | *Ar*  _(_*_i =_* _144)_ | *Ho* | *He* | *PAr* | *Fis* |
| --- | --- | --- | --- | --- | --- | --- | --- |
| Zone 1 | 133 | 9.4 | 8.9 | 0.52 | 0.57 | 0.78 | 0.10 |
| Zone 2 | 109 | 9.3 | 8.7 | 0.56 | 0.58 | 0.82 | 0.02 |
| Zone 3 | 129 | 9.5 | 8.8 | 0.57 | 0.58 | 0.80 | 0.03 |
