# Supplementary material for: Genetic and phenotypic variation along an ecological gradient in lake trout Salvelinus namaycush
Source: BMC Evol Biol. 2016 Oct 19;16:219. doi: 10.1186/s12862-016-0788-8 (PMC5069848; doi:10.1186/s12862-016-0788-8)
Supplement: Additional file 5: — First and second principal coordinate (PC) scores (PC1 and PC2) of an individual-based PC analysis on ecotype genotype. Populations were grouped by approximate confidence ellipses for each ecotype (lean = black circles; humper = light grey circles; siscowet = dark grey circles; redfin = red circles). (DOCX 67 kb) [file 12862_2016_788_MOESM5_ESM.docx]

**Additional file 5**. First and second principal coordinate (PC) scores (*PC*1 and *PC*2) of an individual-based PC analysis on ecotype genotype. Populations were grouped by approximate confidence ellipses for each ecotype (lean = black circles; humper = light grey circles; siscowet = dark grey circles; redfin = red circles).

PC2 (3.4%)

PC1 (4.2%)
