# Supplementary material for: Genetic and phenotypic variation along an ecological gradient in lake trout Salvelinus namaycush
Source: BMC Evol Biol. 2016 Oct 19;16:219. doi: 10.1186/s12862-016-0788-8 (PMC5069848; doi:10.1186/s12862-016-0788-8)

**Additional file 6.** Bayesian genetic population structure barplots for *K* = 2 to *K* = 4 (top) as implemented in program STRUCTURE using 15 loci for Isle Royale lake trout populations divided by three zones and three water depth strata. The light blue, medium blue, and dark blue bands above the plots indicate Zone 1, Zone 2, and Zone 3. Plots of the mean of estimated natural log probably of *K* [*L(K)*] (bottom left) and delta K (∆*K*) to determine number of populations (*K*) (bottom right) were made using the Evanno in program STRUCTURE HARVESTER Web 0.6.92.


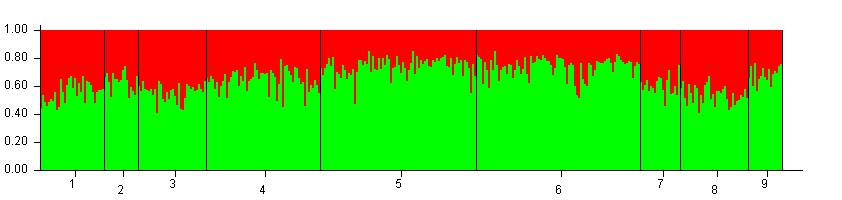
K = 2


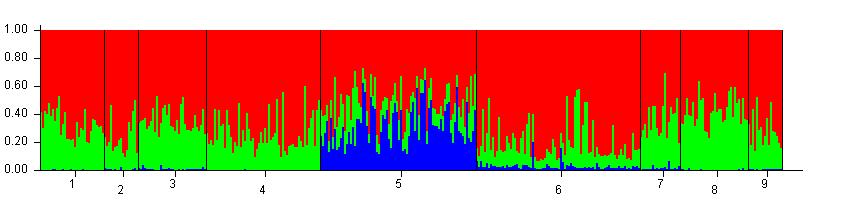
K = 3
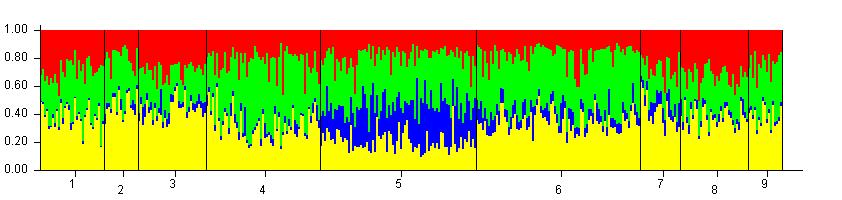
 K = 4

Stratum 2

50 – 100 m

Stratum 1

<50 m

Stratum 3

>100 m


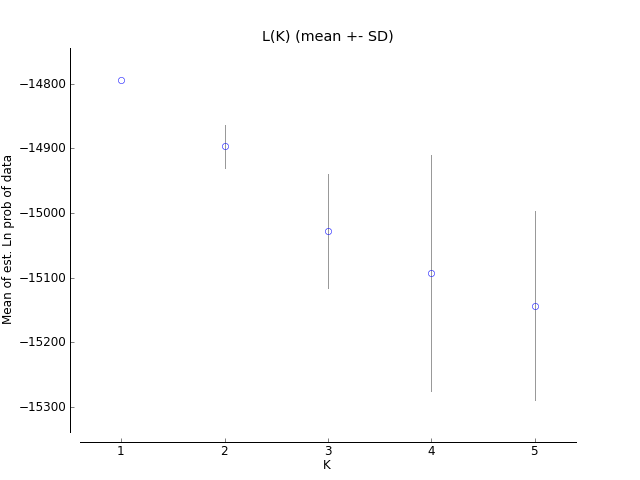

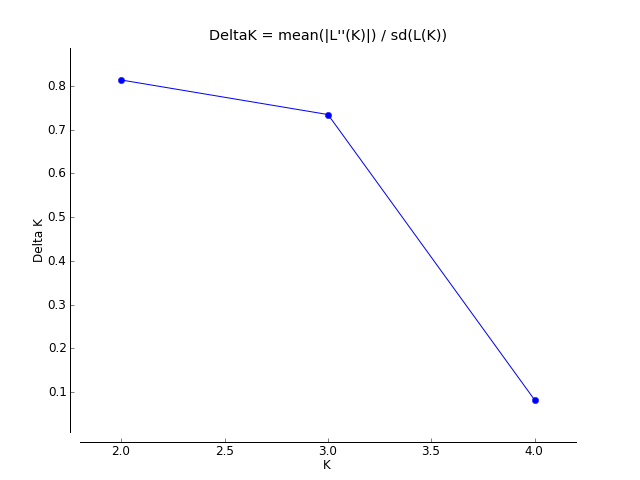

Supplement: Additional file 6: — Bayesian genetic population structure barplots for K = 2 to K = 4 (top) as implemented in program STRUCTURE using 15 loci for Isle Royale lake trout populations divided by three zones and three water depth strata. The light blue, medium blue, and dark blue bands above the plots indicate Zone 1, Zone 2, and Zone 3. Plots of the mean of estimated natural log probably of K [L(K)] (bottom left) and delta K (∆K) to determine number of populations (K) (bottom right) were made using the Evanno in program STRUCTURE HARVESTER Web 0.6.92. (DOCX 158 kb) [file 12862_2016_788_MOESM6_ESM.docx]
