# Supplementary material for: Genetic and phenotypic variation along an ecological gradient in lake trout Salvelinus namaycush
Source: BMC Evol Biol. 2016 Oct 19;16:219. doi: 10.1186/s12862-016-0788-8 (PMC5069848; doi:10.1186/s12862-016-0788-8)
Supplement: Additional file 7: — Membership coefficients (Q; computed using STRUCTURE) at K = 2 to K = 4 group clusters for Isle Royale lake trout genotypes divided by from three water depth strata (<50 m, 50–100 m, >100 m) and three zones. The highest Q scores for likelihood of membership for each inferred cluster, QI to QIV, are shaded in grey. (DOCX 22 kb) [file 12862_2016_788_MOESM7_ESM.docx]

**Additional file 7.** Membership coefficients (*Q*; computed using STRUCTURE) at *K* = 2 to *K* = 4 population clusters for Isle Royale lake trout genotypes divided by from three water depth strata (<50 m, 50 – 100 m, >100 m) and three zones. The highest *Q* scores for likelihood of membership for each inferred cluster, *Q*I to *Q*IV, are shaded in grey.

|  | *K* = 2 |  | *K* = 3 |  |  | *K* = 4 |  |  |  |
| --- | --- | --- | --- | --- | --- | --- | --- | --- | --- |
| Given population | *Q*I | *Q*II | *Q*I | *Q*II | *Q*III | *Q*I | *Q*II | *Q*III | *Q*IV |
| Zone 1 Strata 1 | 0.371 | 0.629 | 0.324 | 0.021 | 0.654 | 0.296 | 0.306 | 0.382 | 0.015 |
| Zone 2 Strata 1 | 0.301 | 0.699 | 0.237 | 0.039 | 0.724 | 0.195 | 0.300 | 0.473 | 0.032 |
| Zone 3 Strata 1 | 0.378 | 0.622 | 0.341 | 0.054 | 0.605 | 0.294 | 0.211 | 0.445 | 0.051 |
| Zone 1 Strata 2 | 0.302 | 0.698 | 0.263 | 0.054 | 0.683 | 0.227 | 0.399 | 0.334 | 0.039 |
| Zone 2 Strata 2 | 0.232 | 0.768 | 0.190 | 0.304 | 0.506 | 0.192 | 0.338 | 0.219 | 0.251 |
| Zone 3 Strata 2 | 0.215 | 0.785 | 0.179 | 0.101 | 0.720 | 0.173 | 0.385 | 0.362 | 0.080 |
| Zone 1 Strata 3 | 0.349 | 0.651 | 0.309 | 0.093 | 0.598 | 0.277 | 0.199 | 0.436 | 0.087 |
| Zone 2 Strata 3 | 0.412 | 0.588 | 0.377 | 0.043 | 0.580 | 0.328 | 0.244 | 0.388 | 0.039 |
| Zone 3 Strata 3 | 0.283 | 0.717 | 0.261 | 0.032 | 0.707 | 0.244 | 0.323 | 0.409 | 0.023 |
