# Supplementary material for: Genetic and phenotypic variation along an ecological gradient in lake trout Salvelinus namaycush
Source: BMC Evol Biol. 2016 Oct 19;16:219. doi: 10.1186/s12862-016-0788-8 (PMC5069848; doi:10.1186/s12862-016-0788-8)
Supplement: Additional file 8: — Mantel test results as implemented in GENALEX [62] on matrix correlation (rm) between genetic distance and water depth (a and b), and genetic distance and geographic distance (c). The relationship shown in graph ‘a’ was significant after interpolation of the data point highlighted. The original non-significant relationship and outlier data point are shown in graph ‘b’. (DOCX 93 kb) [file 12862_2016_788_MOESM8_ESM.docx]

**Additional file 8.** Mantel test results as implemented in GENALEX [62] on matrix correlation (*r*_m_) between genetic distance and water depth (a and b), and genetic distance and geographic distance (c). The relationship shown in graph ‘a’ was significant after interpolation of the data point highlighted. The original non-significant relationship and outlier data point are shown in graph ‘b’.

a) b)

Genetic distance (*Fst*/(1 – *Fst*)

Genetic distance (*Fst*/(1 – *Fst*)


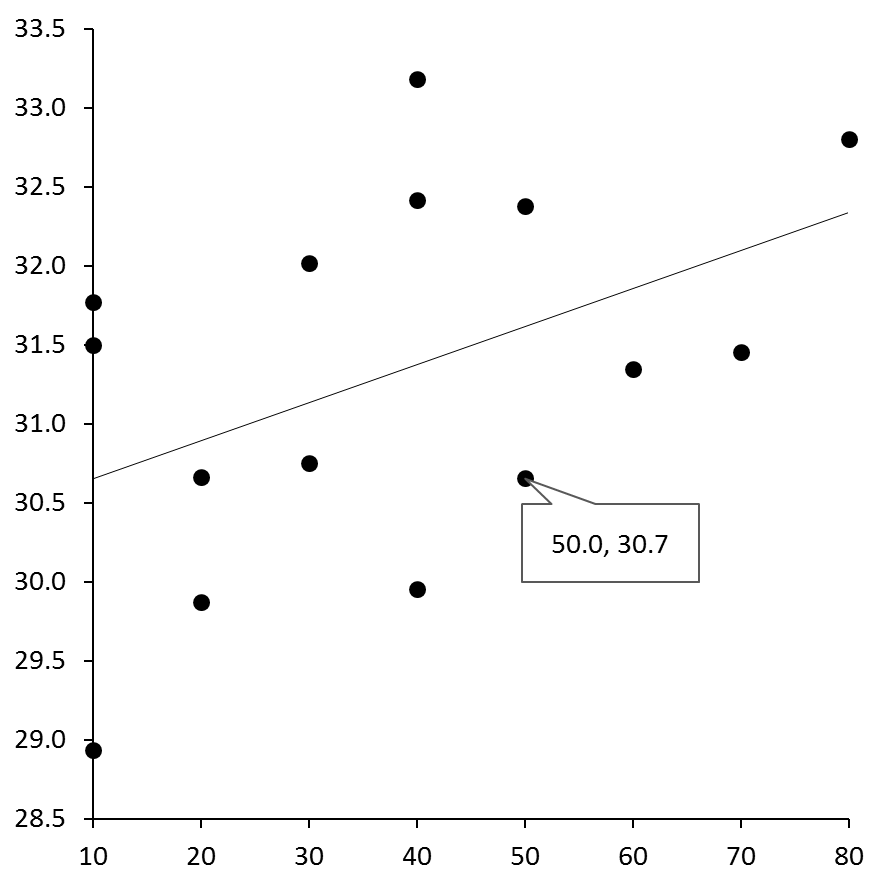

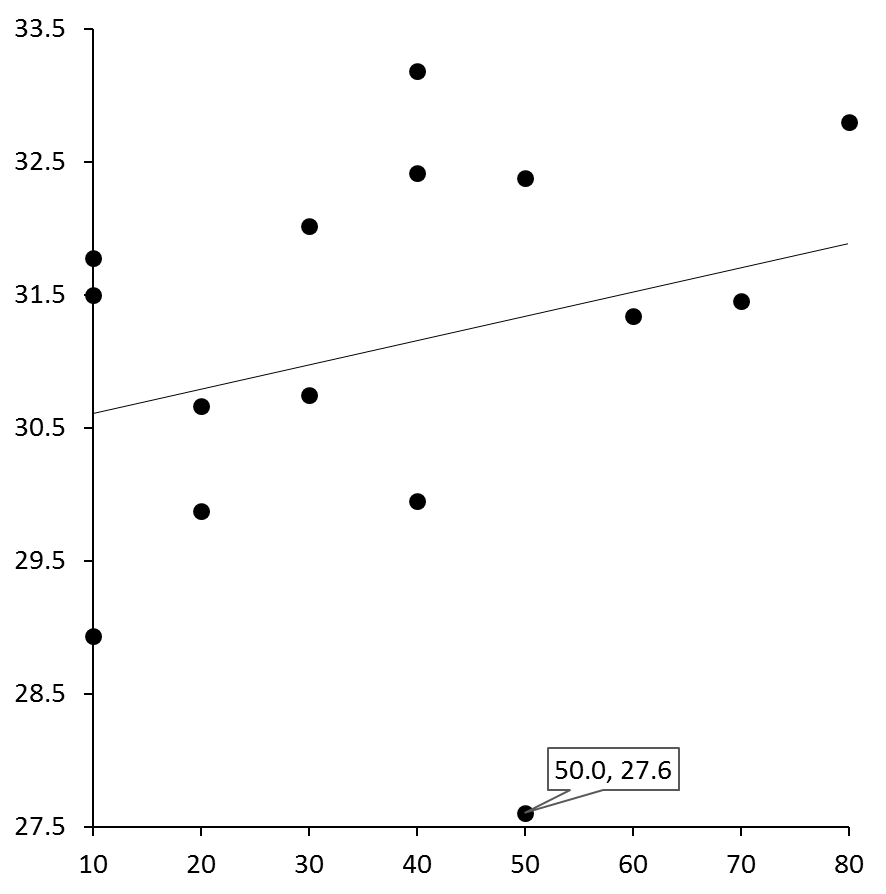


*r*_m_ = 0.26, *P* = 0.217

*r*_m_ = 0.45, *P* = 0.047

Water depth (in metres)

Water depth (in metres)

c)

Genetic distance (*Fst*/(1 – *Fst*)


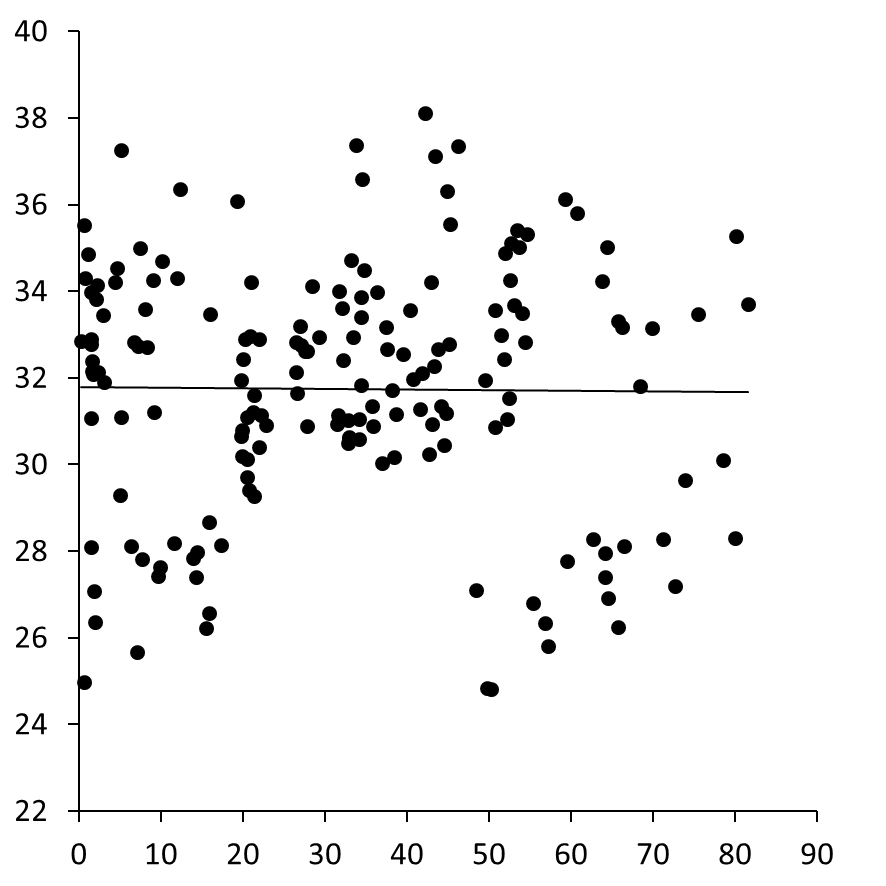


*r*_m_ = -0.05, *P* = 0.7

Geographic distance (in thousands of metres)
