# Supplementary material for: Genetic and phenotypic variation along an ecological gradient in lake trout Salvelinus namaycush
Source: BMC Evol Biol. 2016 Oct 19;16:219. doi: 10.1186/s12862-016-0788-8 (PMC5069848; doi:10.1186/s12862-016-0788-8)
Supplement: Additional file 9: — Within-group phenotypic variance based on diagonal of the R matrix model and standard error as calculated in program RMET 5.0 [76–78]. (DOCX 22 kb) [file 12862_2016_788_MOESM9_ESM.docx]

**Additional file 9.** Within-group phenotypic variance (*r*ii) based on diagonal of the *R* matrix model and standard error as calculated in program RMET 5.0 [76-78].

| phenotype | < 50 m | 50 - 100 m | > 100 m |
| --- | --- | --- | --- |
| Body shape† | 0.075 ± 0.043 (84) | 0.089 ± 0.089 (200) | 0.000 ± 0.003 (53) |
| Head shape† | 0.047 ± 0.035 (83) | 0.056 ± 0.025 (187) | 0.000 ± 0.003 (50) |
| Buoyancy | 0.124 ± 0.052 (83) | 0.006 ± 0.009 (196) | 0.198 ± 0.085 (51) |
| Pectoral fin length | 0.000 ± 0.004 (84) | 0.001 ± 0.006 (181) | 0.000 ± 0.007 (51) |
| Pelvic fin length* | 0.000 ± 0.008 (84) | 0.045 ± 0.023 (181) | 0.068 ± 0.054 (51) |
| Caudal peduncle depth | 0.028 ± 0.028 (84) | 0.000 ± 0.001 (181) | 0.029 ± 0.039 (51) |
| Caudal peduncle length† | 0.027 ± 0.027 (84) | 0.043 ± 0.022 (181) | 0.000 ± 0.006 (51) |
| Maxilla length | 0.008 ± 0.018 (84) | 0.002 ± 0.007 (181) | 0.026 ± 0.037 (51) |
| Orbital length | 0.000 ± 0.001 (84) | 0.010 ± 0.012 (181) | 0.004 ± 0.023 (51) |
| Preorbital length | 0.002 ± 0.013 (84) | 0.000 ± 0.005 (181) | 0.000 ± 0.007 (51) |
| Head length | 0.000 ± 0.006 (84) | 0.000 ± 0.002 (181) | 0.000 ± 0.012 (51) |
| Age at length zero (t0) | 0.025 ± 0.028 (77) | 0.014 ± 0.013 (191) | 0.000 ± 0.009 (54) |
| Length at age zero (L0) | 0.010 ± 0.021 (77) | 0.009 ± 0.011 (191) | 0.000 ± 0.004 (54) |
| Early growth rate (Ω) | 0.0107 ± 0.053 (77) | 0.033 ± 0.019 (191) | 0.012 ± 0.027 (54) |
| Instantaneous growth rate (K) | 0.000 ± 0.006 (77) | 0.000 ± 0.007 (191) | 0.000 ± 0.006 (54) |
| Asymptotic length (Lmax)† | 0.231 ± 0.074 (77) | 0.070 ± 0.026 (191) | 0.038 ± 0.039 (54) |

* Significant increase in variance with depth.

† Significant decrease in variance with depth.
